# An investigation of penalization and data augmentation to improve convergence of generalized estimating equations for clustered binary outcomes

**DOI:** 10.1186/s12874-022-01641-6

**Published:** 2022-06-09

**Authors:** Angelika Geroldinger, Rok Blagus, Helen Ogden, Georg Heinze

**Affiliations:** 1 Medical University of Vienna, Center for Medical Statistics, Informatics and Intelligent System, Section for Clinical Biometrics, Spitalgasse 23, A-1090 Vienna, Austria; 2 University of Ljubljana, Institute of Biostatistics and Medical Informatics, Ljubljana, Slovenia; 3 University of Southampton, School of Mathematical Sciences, Southampton, UK

**Keywords:** Clustered data, Firth’s logistic regression, Generalized estimating equations, Logistic regression, Non-convergence, Separation

## Abstract

**Background:**

In binary logistic regression data are ‘separable’ if there exists a linear combination of explanatory variables which perfectly predicts the observed outcome, leading to non-existence of some of the maximum likelihood coefficient estimates. A popular solution to obtain finite estimates even with separable data is Firth’s logistic regression (FL), which was originally proposed to reduce the bias in coefficient estimates. The question of convergence becomes more involved when analyzing clustered data as frequently encountered in clinical research, e.g. data collected in several study centers or when individuals contribute multiple observations, using marginal logistic regression models fitted by generalized estimating equations (GEE). From our experience we suspect that separable data are a sufficient, but not a necessary condition for non-convergence of GEE. Thus, we expect that generalizations of approaches that can handle separable uncorrelated data may reduce but not fully remove the non-convergence issues of GEE.

**Methods:**

We investigate one recently proposed and two new extensions of FL to GEE. With ‘penalized GEE’ the GEE are treated as score equations, i.e. as derivatives of a log-likelihood set to zero, which are then modified as in FL. We introduce two approaches motivated by the equivalence of FL and maximum likelihood estimation with iteratively augmented data. Specifically, we consider fully iterated and single-step versions of this ‘augmented GEE’ approach. We compare the three approaches with respect to convergence behavior, practical applicability and performance using simulated data and a real data example.

**Results:**

Our simulations indicate that all three extensions of FL to GEE substantially improve convergence compared to ordinary GEE, while showing a similar or even better performance in terms of accuracy of coefficient estimates and predictions. Penalized GEE often slightly outperforms the augmented GEE approaches, but this comes at the cost of a higher burden of implementation.

**Conclusions:**

When fitting marginal logistic regression models using GEE on sparse data we recommend to apply penalized GEE if one has access to a suitable software implementation and single-step augmented GEE otherwise.

**Supplementary Information:**

The online version contains supplementary material available at 10.1186/s12874-022-01641-6.

## Introduction

When modeling a binary outcome with a set of explanatory variables using logistic regression, one frequently encounters the problem of separation. With separable data a linear combination of explanatory variables perfectly predicts the observed outcomes, and then some of the regression coefficients do not exist and their estimates diverge during the iterative fitting process [1]. The occurrence of separation is prevalent with unbalanced outcomes or binary covariates, small sample sizes and strong effects. One possibility to obtain finite estimates of regression coefficients even in the case of separation is to resort to Firth’s logistic regression (FL), which was originally proposed to reduce the bias in coefficient estimates compared to maximum likelihood estimation [2]. The question of convergence becomes more involved when we want to model clustered data, as frequently encountered in clinical research, e.g. data collected in several study centers or when individuals contribute multiple observations. With such multilevel data one has to decide whether the research question is better addressed by fitting a marginal or a conditional model, depending on the level of sampling (the clusters or the observations) that is of main interest. In this paper, we will only deal with marginal logistic regression models fitted by generalized estimating equations (GEE). Lacking a formal proof, our experience suggests that separable data are a sufficient, but not a necessary condition for non-convergence of GEE. Therefore, extensions of approaches that can deal with separation in uncorrelated data may not fully remove all non-convergence issues in the setting of GEE. Still, they may considerably improve on ordinary GEE.

As FL efficiently solves estimation problems with separable uncorrelated data, we investigated one recently proposed and two new extensions of FL to GEE. While Paul and Zhang [3] as well as Mondol and Rahman [4] proposed to treat GEE as score equations, i.e. as derivatives of a log-likelihood set to zero, which are then modified as in FL, we will introduce some approaches which are motivated by the equivalence of FL and maximum likelihood estimation with iteratively augmented data. Specifically, we will consider fully iterated and single-step versions of these data augmentation procedures, and we will compare these extensions with respect to convergence behavior, practical applicability and performance in terms of estimation and prediction using simulated data. While FL was shown to give coefficient estimates of smaller bias than maximum likelihood estimation, it is beyond the scope of this paper to theoretically investigate similar properties for the three extensions of FL to GEE. Nevertheless, the following considerations justify the investigation of the methods:

- all three approaches generalize FL in the sense that they give the same coefficient estimates as FL in the special situation of an independent working correlation, where the GEE can be interpreted as score equations,
- all three approaches improve on ordinary GEE in terms of convergence and accuracy of coefficient and prediction estimates in our simulations,
- penalized GEE is a published method pending independent evaluation by the scientific community,
- under independence, all satisfactory solutions that preserve transformation invariance and do not need additional parameter tuning are based on the Jeffreys prior/Firth penalty.

In the next section we will discuss the issue of separation, review FL and GEE and introduce the approaches to extend FL to GEE. The subsequent section illustrates the application of these methods on data from a study in implant dentistry. Next, the methods are compared in a simulation study with clustered data. Finally, we summarize our findings, discuss possible extensions and give recommendations for practical applications.

## Methods

### Separation and Firth’s logistic regression

Logistic regression models the probability that *Y*_*i*_ = 1 for independent binary outcomes *Y*_*i*_, *i* = 1, …*N*, with values *y*_*i*_ ∈ {0, 1}, given (*p* + 1)-dimensional row vectors of covariate values *x*_*i*_ = (1, *x*_*i*1_, …, *x*_*ip*_) by assuming

$$P\left({Y}_i=1|{x}_i\right)={\left(1+\exp \left(-{x}_i\beta \right)\right)}^{-1}={\pi}_i\left(\beta \right),$$

where *β* = (*β*_0_, *β*_1_, …, *β*_*p*_)^′^ is a vector of regression coefficients. Maximum likelihood estimates for *β* can be obtained by solving the score equations. Albert and Anderson found that finite maximum likelihood estimates exist if and only if the data are not separable, i.e. if there exists no hyperplane defined by a linear combination of covariates that separates events and non-events [1]. There are two main drivers for the occurrence of separation: the presence of strong effects and a low amount of information available in the data, manifested, e.g., by a small sample size or rare occurrence of one of the two levels of the outcome variable or of a binary covariate. FL, which originally was proposed to reduce the bias of maximum likelihood estimates [2], has been propagated as a reliable alternative to maximum likelihood estimation [5] as it always provides finite coefficient estimates. With FL, the likelihood is penalized by the Jeffreys invariant prior, i.e. by the square root of the determinant of the Fisher information matrix |*I*(*β*)|^1/2^. On the level of score eqs. FL adds 1/2 trace(*I*(*β*)^−1^ (*∂I*(*β*)/*∂β*)) to the first derivative of the log-likelihood. By proving that the logarithm of the Jeffreys prior $\frac{1}{2}\ \log \left(I\left(\beta \right)\right)$ tends to −∞ if one of the components of *β* approaches ∞, Kosmidis and Firth showed that the penalized log likelihood is always maximized by finite *β*, i.e. FL always provides finite coefficient estimates [6]. Another convenient property of FL, which is for instance not shared by other proposals for penalized likelihood logistic regression, is that FL is transformation invariant. This means that if *G* is an invertible (*p* + 1) × (*p* + 1)-matrix, ${\hat{\beta}}_X$ the FL coefficient estimate for the design matrix *X* with rows *x*_*i*_, *i* = 1, …, *N*, and ${\hat{\beta}}_{XG}$ the FL coefficient estimate for the design matrix *X* · *G*, then we have ${\hat{\beta}}_{XG}={G}^{-1}\cdot {\hat{\beta}}_X$.

FL is equivalent to maximum likelihood estimation with an appropriately augmented and weighted data set $\left(\overset{\sim }{y},\overset{\sim }{X}\right)$ containing 3*N* observations, basically obtained by stacking three copies of the original data set [7]. For *j* ∈ {1, …, 3*N*} we denote by *i*_*j*_ the integer in {1, …, *N*} such that *j* ∈ {*i*_*j*_, *N* + *i*_*j*_, 2*N* + *i*_*j*_}. The covariate row vectors $\tilde{x}_j,$ outcomes $\tilde{y}_j$ and the weights $\tilde{w}_j$, *j* ∈ {1, …, 3*N*}, of the augmented data set $\left(\overset{\sim }{y},\overset{\sim }{X}\right)$ are defined by $\tilde{x}_j:= {x}_{i_j},$

$$\tilde{y}_j:= \left\{\begin{array}{c}{y}_{i_j},\kern0.5em \mathrm{if}\ j={i}_j\ \mathrm{or}\ j=N+{i}_j,\\ {}1-{y}_{i_j},\kern0.5em \mathrm{if}\ j=2N+{i}_j\end{array}\right.$$

and

$$\tilde{w}_j:= \left\{\begin{array}{c}1,\kern0.5em \mathrm{if}\ j={i}_j,\\ {}{h}_{i_j}/2,\kern0.5em \mathrm{if}\ j=N+{i}_j\ \mathrm{or}\ j=2N+{i}_j,\end{array}\right.$$

where *h*_*i*_ denotes the *i*-th diagonal element of the hat matrix $\overset{\sim }{H}={W}^{1/2}X\ {\left({X}^{\prime } WX\right)}^{-1}{X}^{\prime }{W}^{1/2}$, with *X* the *N* × (*p* + 1)- design matrix and *W* the diagonal matrix with elements *π*_*i*_(1 − *π*_*i*_).

Since the contribution of the data augmentation is asymptotically negligible, approximate standard errors of the estimates can be obtained as the square roots of the diagonal elements of (*X*^′^*WX*)^−1^, where the elements ${\hat{\pi}}_i\left(1-{\hat{\pi}}_i\right)$ in the *N* × *N* matrix *W* are obtained using the coefficient estimates from the final iteration.

### Generalized estimating equations

We will now slightly adapt our notation to the situation of clustered data. Let *N* be the number of clusters and let *n*_*i*_ be the number of observations in the *i*-th cluster. We denote by ${y}_i={\left({y}_{i1},\dots, {y}_{in_i}\right)}^{\prime }$ the *n*_*i*_-dimensional vector of binary outcomes in the *i*-th cluster, by *x*_*ij*_ = (1, *x*_*ij*1_, …, *x*_*ijp*_)^′^ the (*p* + 1)-dimensional vector of covariates for the *j*-th observation in the *i*-th cluster and by *X*_*i*_ the (*n*_*i*_ × (*p* + 1))-dimensional matrix with rows *x*_*ij*_, *i* = 1, …, *N* and *j* = 1, …, *n*_*i*_. The marginal logistic regression model relates the conditional expectation *P*(*Y*_*ij*_ = 1| *x*_*ij*_) to the vector of covariates by assuming *P*(*Y*_*ij*_ = 1| *x*_*ij*_) = (1 + exp(*x*_*ij*_*β*))^−1^ for a (*p* + 1)-dimensional vector of parameters *β* = (*β*_0_, *β*_1_, …, *β*_*p*_)^′^ [8]. Using a working correlation structure expressed as a (*n*_*i*_ × *n*_*i*_)-dimensional matrix *R*_*i*_(*α*) with some parameter vector *α*, Liang and Zeger [9] showed that the parameters *β* can be consistently estimated by solving the generalized estimating equations

$$\sum_{i=1}^N{X_i}^{\prime }{W}_i\ {\left({W}_i^{1/2}{R}_i\left(\alpha \right){W}_i^{1/2}\right)}^{-1}\left({y}_i-{\pi}_i\right)=0,\kern0.5em (1)$$

where *π*_*i*_ is the *n*_*i*_-dimensional vector with components *π*_*ij*_ = (1 + exp(*x*_*ij*_*β*))^−1^ and *W*_*i*_ is the (*n*_*i*_ × *n*_*i*_)-dimensional diagonal matrix with elements π_*ij*_(1 − *π*_*ij*_). Assuming an independent working correlation, i.e. setting *R*_*i*_(*α*) to the identity matrix, the estimating eqs. (1) reduce to the score equations for logistic regression with independent observations.

### Transferring Firth’s likelihood penalization to generalized estimating equations

The considerations on FL above imply three possible extensions of FL to GEE.

#### Penalized GEE (penGEE)

First, considering GEE as score equations leads to the following penalized GEE

$${\displaystyle \begin{array}{c}{U}^{\ast}\left(\beta, \alpha \right)=U\left(\beta, \alpha \right)+\frac{1}{2}\mathrm{trace}\ \left(I{\left(\beta, \alpha \right)}^{-1}\left(\frac{\partial I\left(\beta, \alpha \right)}{\partial \beta}\right)\right)=0, (2)\end{array}}$$

where *U*(*β*, *α*) is the generalized estimating function given by the left hand side of eq. (1) and *I*(*β*, *α*)= $-E\left(\frac{\partial U\left(\beta, \alpha \right)}{\partial \beta}\right)$[3, 4]. As with ordinary GEE, a solution to eq. (2) can be obtained by iterating between solving for *β* by the first step of a Newton-Raphson fitting algorithm given a value *α*, and updating *α* using the method of moments-based estimators. This motivates the following ‘penalized GEE’ algorithm:

1. As initial estimate ${\hat{\beta}}^0$ for *β* use the coefficient estimate obtained by applying FL to the data ignoring the clustering structure.
2. Given the estimate ${\hat{\beta}}^k$ for *β*, *k* ∈ *ℕ*, calculate the moment-based estimate ${\hat{\alpha}}^{k+1}$ for *α*. For example, in the setting of an exchangeable working correlation structure set
  $${\hat{\alpha}}^{k+1}=\frac{1}{N}\sum_{i=1}^N\frac{1}{n_i\left({n}_i-1\right)}\sum_{j\ne k}^{n_i}{\hat{e}}_{ij}{\hat{e}}_{ik},$$

where ${\hat{e}}_{ij}=\left({y}_{ij}-{\hat{\pi}}_{ij}\right)/\sqrt{\left({\hat{\pi}}_{ij}\left(1-{\hat{\pi}}_{ij}\right)\right)}$. See Molenberghs and Verbeke [10] (p.157) for the formulas for independent, AR(1) and unstructured working correlation structure.

3. Update the coefficient estimate by
  $${\hat{\beta}}^{k+1}={\hat{\beta}}^k+I{\left({\hat{\beta}}^k,{\hat{\alpha}}^k\right)}^{-1}\cdot {U}^{\ast}\left({\hat{\beta}}^k,{\hat{\alpha}}^k\right).$$
4. Repeat steps 2 and 3 until convergence is achieved.

Note that penalized GEE with independent working correlation structure results in the same coefficient estimates as FL.

#### Iterated augmented GEE (augGEE)

Second, we suggest extending FL to GEE by mimicking the data augmentation approach. The idea is to iterate between augmenting the data given the current estimates of *β* and *α*, and re-solving the GEE using the augmented data to obtain new estimates. This gives rise to the following ‘iterated augmented GEE’ algorithm:

1. As initial estimate ${\hat{\beta}}^0$ for *β* use the coefficient estimate obtained by applying FL to the data ignoring the clustering structure. As initial estimate ${\hat{\alpha}}^0$ for *α* use the value corresponding to a working correlation structure $R\left({\hat{\alpha}}^0\right)$ equal to the identity matrix.
2. Given the current estimate ${\hat{\beta}}^k$ of *β* and ${\hat{\alpha}}^k$ of *α* for *k* ∈ *ℕ*, calculate the block diagonal matrix ${H}^k=\operatorname{diag}\left({H}_1^k,\dots, {H}_N^k\right)$ with blocks ${H}_i^k={\Omega}_{\mathrm{i}}^{1/2}{X}_i{\left({X}_i^{\prime}\Omega {X}_i\right)}^{-1}{X}_i^{\prime }{\Omega}_{\mathrm{i}}^{1/2}$, where ${\Omega}_{\mathrm{i}}={W}_i^{1/2}{R}_i{\left({\hat{\alpha}}^k\right)}^{-1}{W}_i^{1/2}$[11]. This block diagonal matrix generalizes the hat matrix $\overset{\sim }{H}$ defined for independent data in Section 2.1.
3. Similarly as in Section 2.1 create an augmented data set $\left(\overset{\sim }{y},\overset{\sim }{X}\right)$, consisting of $\overset{\sim }{N}=3N$ clusters with $\tilde{n}_l={n}_{i_l}$ observations in the *l*-th cluster, where *i*_*l*_ is the integer in {1, …, *N* } such that *l* ∈ {*i*_*l*_, *N* + *i*_*l*_, 2*N* + *i*_*l*_}. The vector of covariates $\tilde{x}_{lj},l=1,\dots, \overset{\sim }{N},j=1,\dots, \tilde{n}_l,$ for the *j*-th observation in the *l*-th cluster in the augmented data is set to ${x}_{i_lj}$. The outcome $\tilde{y}_{lj}$ and the weights $\tilde{w}_{lj}$ for this augmented data set are given by
  $$\tilde{y}_{lj}=\left\{\begin{array}{c}{y}_{i_lj},\kern0.5em \mathrm{if}\ l={i}_l\ \mathrm{or}\ l=N+{i}_l,\\ {}1-{y}_{i_lj},\kern0.5em \mathrm{if}\ l=2N+{i}_l\end{array}\right.$$

and

$$\tilde{w}_{lj}=\left\{\begin{array}{c}1,\kern0.5em \mathrm{if}\ l={i}_l,\\ {}{h}_{i_lj}/2,\kern0.5em \mathrm{if}\ l=N+{i}_l\ \mathrm{or}\ l=2N+{i}_l,\end{array}\right.$$

where *h*_*ij*_ denotes the *j*-th diagonal element of the *i*-th block ${H}_i^k$ of *H*^*k*^.

4. Solve the GEE on the augmented data set $\left(\overset{\sim }{y},\overset{\sim }{X}\right)$. Set ${\hat{\beta}}^{k+1}$ to the coefficient estimate and ${\hat{\alpha}}^{k+1}$ to the estimated parameter for the working correlation structure.
5. Repeat steps 2, 3 and 4 until convergence is achieved. The final coefficient estimate $\hat{\beta}$ and correlation parameter estimate $\hat{\alpha}$ are the estimates from the last iteration.

In step 3 of the iterated augmented GEE algorithm one might think of alternative ways of defining the clustering structure for the augmented data set $\left(\overset{\sim }{y},\overset{\sim }{X}\ \right)$. For instance, one could combine the *i*-th, (*N* + *i*)-th and (2*N* + *i*)-th clusters, *i* ∈ {1, …, *N*}, resulting in an augmented data set consisting of only *N* instead of 3*N* clusters. However, creating two ‘pseudo clusters’ for each original cluster as suggested in the iterated augmented GEE algorithm best reflects the data augmentation approach in the situation of independent data, where the pseudo observations are also treated as independent observations representing the Jeffreys prior.

As the trace of the generalized hat matrix *H* calculated in step 2 of the iterated augmented GEE algorithm is always equal to *p* + 1 [11], i.e. the total weight of the ‘pseudo observations’ is *p* + 1, we conclude that the relative contribution of the pseudo observations becomes negligible with increasing sample size and that the algorithm yields consistent estimates.

#### Single-step augmented GEE (augGEE1)

Third, we investigated a simpler version of the augmented GEE algorithm which can be easily implemented whenever fitting algorithms for FL and weighted GEE are available. This ‘single-step augmented GEE’ algorithm consists of the following steps:

1. Apply FL to the data ignoring the clustering and construct the corresponding augmented data set $\left(\overset{\sim }{y},\overset{\sim }{X}\right)$ as described in section 2.1, i.e. using the ordinary hat matrix $\overset{\sim }{H}$ in defining the weights, not the hat matrix *H*^*k*^ as in the iterated augmented GEE algorithm.

2. Define the clustering on $\left(\overset{\sim }{y},\overset{\sim }{X}\right)$ by treating each of the three copies of the original data as independent data sets, similarly as in the iterated augmented GEE algorithm. In this way, the augmented data $\left(\overset{\sim }{y},\overset{\sim }{X}\right)$ contains 3*N* (clusters)

3. Solve the GEE on the augmented data set $\left(\overset{\sim }{y},\overset{\sim }{X}\right)$, this gives the final regression coefficient estimate$\hat{\beta}$ and correlation parameter estimate $\hat{\alpha}$.

In other words, single-step augmented GEE can be performed by stopping the iterated augmented GEE algorithm after the first outer iteration when using the ordinary hat matrix $\overset{\sim }{H}$ instead of *H*^0^. Note that applying iterated or single-step augmented GEE with an independent working correlation structure gives the same coefficient estimates as FL. The consistency of the single-step augmented GEE can be shown analogously to the consistency of the iterated algorithm.

### Estimating the variance-covariance matrix

With GEE a consistent estimate of the asymptotic variance-covariance matrix of the regression coefficients is given by the so-called sandwich estimate

$$S\left(\hat{\beta}\right)={I}_0{\left(\hat{\beta}\right)}^{-1}\ {I}_1\left(\hat{\beta}\right){I}_0{\left(\hat{\beta}\right)}^{-1},$$

where

$${I}_0\left(\hat{\beta}\right)=\sum_{i=1}^N{X}_i^{\prime }{\hat{W}}_i\ {\hat{V}}_i^{-1}{\hat{W}}_i{X}_i,\kern7.75em {I}_1\left(\hat{\beta}\right)=\sum_{i=1}^N{d}_i{d}_i^{\prime },$$

${d}_i={X}_i^{\prime }{\hat{W}}_i{\hat{V}}_i^{-1}\left({y}_i-{\hat{\uppi}}_i\right)$ and ${V}_i={W}_i^{1/2}{R}_i\left(\alpha \right){W}_i^{1/2}$ is the working variance-covariance matrix [10]. With small samples, the sandwich estimator is known to underestimate the variance [12]. We will use the small-sample correction proposed by Morel et al. [13] to correct for this underestimation. The corrected variance-covariance matrix ${S}^{\ast}\left(\hat{\beta}\right)$ is defined as

$${S}^{\ast}\left(\hat{\beta}\right)={I}_0{\left(\hat{\beta}\right)}^{-1}\ {I}_1^{\ast}\left(\hat{\beta}\right){I}_0{\left(\hat{\beta}\right)}^{-1}+\delta \cdot \phi \cdot {I}_0{\left(\hat{\beta}\right)}^{-1}$$

with

$${I}_1^{\ast}\left(\hat{\beta}\right)=\frac{N^{\ast }-1}{N^{\ast }-p-1}\cdot \frac{N}{N-1}\cdot \sum_{i=1}^N\left({d}_i-\overline{d}\right){\left({d}_i-\overline{d}\right)}^{\prime },$$

$$\overline{d}=\frac{1}{N}\sum_{i=1}^N{d}_i,$$

N* = ∑_*i*_*n*_*i*_ the number of observations,$\delta =\min \left(0.5,\frac{p+1}{N-p-1}\right)$ and $\phi =\max \left(1,\frac{\mathrm{trace}\left(\alpha \cdot {I}_0{\left(\hat{\beta}\right)}^{-1}{I}_1^{\ast}\left(\hat{\beta}\right)\right)}{p+1}\right)$. We recommend to calculate the variance-covariance matrices for the two augmented GEE approaches by applying the sandwich estimator with small-sample correction to the original data (*y*, *X*) using the parameter estimates $\hat{\beta}$ and $\hat{\alpha}$. Note that this is different from the sandwich estimates of the variance-covariance matrix which come with the last GEE fits in the two augmented GEE algorithms, as there the sandwich estimator is applied to the augmented data set $\left(\overset{\sim }{y},\overset{\sim }{X}\right)$.

Any 95% confidence intervals reported in the following for ordinary GEE, penalized GEE, single-step and iterated augmented GEE were calculated by multiplying the standard error derived from the small-sample corrected sandwich estimates of the variance covariance matrix with the 0.025-th and 0.975-th quantile of the t-distribution with the number of clusters as degree of freedom.

### Implementation

For penalized GEE, we modified the R package ‘geefirthr’ available on GitHub at https://github.com/mhmondol/geefirthr. The version of ‘geefirthr’ available on GitHub cannot handle clusters consisting of single observations and additionally estimates a scale parameter. Our modified version of ‘geefirthr’, which can also deal with clusters containing one observation and with the scale parameter set to 1, can be found at https://github.com/heogden/geefirthr. To implement the single-step and the iterated augmented GEE algorithms, we combined the function logistf in the R package ‘logistf’ version 1.24.1 [14], which implements FL, and the function geem2 in the R package ‘mmmgee’ version 1.20 [15], which implements weighted GEE. One should be aware that there are different ways of implementing weighted GEE. In the function geem2 the weights resemble a scale factor for each observation, similarly as implemented in PROC GEE in SAS^©^ [16]. For the ‘outer loop’ of the iterated augmented GEE approach we used the same convergence criterion as implemented in the R package ‘geefirthr’, i.e. we declared the algorithm as converged if $\underset{\mathrm{m}=0,\dots \mathrm{p}}{\max}\mid {\hat{\beta}}_m^{k+1}-{\hat{\beta}}_m^k\mid <0.001$ within *k* ≤ 20 iterations. We also used this convergence criterion for all GEE fits performed within the two augmented GEE approaches, allowing for 30 iterations. To make the single-step augmented GEE algorithm faster and to facilitate its convergence, we used the FL coefficient estimate obtained in the first step of the algorithm as starting value for the GEE fit performed in the third step. Similarly, with iterated augmented GEE we used the coefficient estimate from the previous iteration as starting value for the GEE fit in the current iteration. R code for the single-step and iterated augmented GEE can be found at https://github.com/AngelikaGeroldinger/augGEE.

## A study on postoperative complications in implant dentistry

We will use data from a clinical study in implant dentistry to illustrate the behavior of GEE and its modifications in the presence of separation and to discuss transformation invariance. The study was set up to investigate the relation of various patient and implant parameters with the occurrence of complications after implant dentistry [17]. For the purpose of illustration we restricted the study data to the 533 implantations in edentulous jaws, performed in 134 subjects. There were 28 haematological complications experienced by only 7 patients, corresponding to an event rate of 0.053, see Fig. S1. We used GEE to associate the occurrence of haematological complications with timing of implant placement (immediate/early/later), diabetes mellitus (yes/no), antiresorptive therapy (yes/no) and age (in decades). Timing of implant placement varies within patients, the other three risk factors are constant within patients. As we do not have information on the date of the implantations, we assumed an exchangeable working correlation structure for all fitted GEE models. The data are separable as all 24 implantations for patients on antiresorptive therapy were performed without complication.

**Fig. 1:**
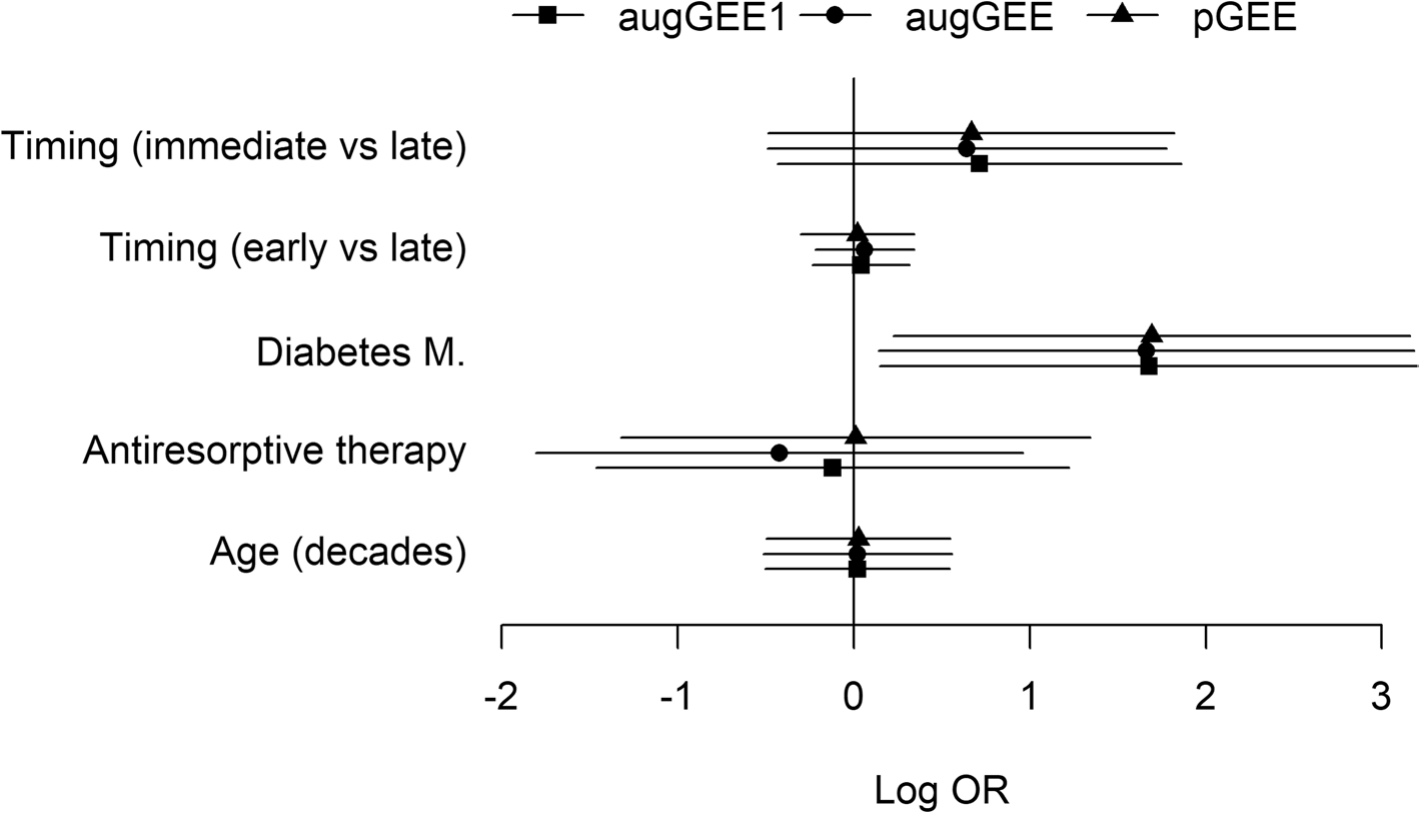
Association of occurrence of haematological complications with timing of implant placement (immediate/early/later), diabetes mellitus (yes/no), antiresorptive therapy (yes/no) and age (in decades) estimated with multivariable single-step augmented generalized estimating equations (augGEE1),iterated augmented GEE (augGEE) and penalized GEE (pGEE). Symbols give the regression coefficients and the lines extend from the lower limits to the upper limits of the 95 % confidence intervals

First, we checked how some of the functions for fitting ordinary GEE in R and SAS deal with non-convergence issues caused by separable data. When we modeled the occurrence of complications with age, diabetes, antiresorptive therapy and timing, four out of the five implementations we investigated (geem in package ‘geeM’ [18], geem2 in ‘mmmgee’ [15], gee in ‘gee’ [19], PROC GEE in SAS software, version 9.4) gave an error message signaling that the fitting algorithm had not converged. Only the function geeglm in the R-package ‘geepack’ [20] did not report an error, leaving it to the user to interpret a regression coefficient of −39.03 for the variable antiresorptive therapy as an indicator for non-convergence of the fitting algorithm. Interestingly, while in logistic regression with independent data separation is usually characterized by large coefficient estimates and large standard errors in the last fitting iteration, the standard error for antiresorptive therapy reported by geeglm was only 0.65 resulting in a very small *p*-value. In contrast, penalized GEE, single-step and iterated augmented GEE gave plausible, finite regression coefficients with reasonable standard errors, see Fig. 1 and Table S1.

Finally, we would like to stress that all three considered modified GEE algorithms inherit the transformation invariance from ordinary GEE, in the sense that changing the scale of a metric variable or the coding of a categorical variable does not alter the conclusions drawn from the analyses. For instance, for the multivariable model described in Fig. 1 and Table S1 single-step augmented GEE returned a regression coefficient of 0.0198 and a standard error of 0.267 for age in decades. If instead age is given in years, single-step augmented GEE yields a coefficient estimate of 0.00198 and a standard error of 0.0267.

## Simulation study

### Set up

We describe the set-up of our simulation study using the framework by Morris et al [21].

#### Aims

First, we aimed to compare the prevalence of non-convergence issues between the methods. Second, we investigated the performance with respect to effect and confidence interval estimation as well as prediction of event probabilities.

#### Data-generating mechanisms

We considered 36 simulation scenarios, varying the number of clusters (*N* ∈ {20, 50, 100}), the size of the clusters (‘small’, ‘moderate’, ‘large’), the strength of the correlation of the binary outcome (‘moderate’, ‘high’) and the event rate ($\overline{y}\in \left\{0.1,0.3\right\}\Big)$, in a full factorial design. For each scenario we created 1000 data sets.

For scenarios with ‘small’ cluster size, we determined the numbers of observations per cluster by sampling from a truncated Poisson distribution with mean 5, minimum 1 and maximum 10. For ‘moderate’ or ‘large’ cluster sizes we used truncated Poisson distributions with mean 10 or 20, minimum 1 and maximum 20 or 40, respectively.

For the generation of the five covariates *X*_1_, …*X*_5_ we followed ideas by Binder et al. [22] in order to obtain realistic data. By first sampling from five standard normally distributed variables *Z*_1_, …*Z*_5_ with correlation structure as defined in Table S2 and then applying the transformations specified in Table S2 we obtained three binary variables *X*_1_, *X*_2_, *X*_3_, one ordinal variable *X*_4_ and one continuous variable *X*_5_. The covariates *X*_1_ and *X*_2_ were generated as ‘between-cluster’ covariates by requiring *Z*_1_ and *Z*_2_ to be constant within clusters, while the three other covariates were generated as ‘within-cluster’ covariates, i.e. they were allowed to vary between the observations of a cluster. To avoid extreme values in the metric covariate *X*_5_, we winsorized it at the third quartile plus three times the interquartile range and at the first quartile minus three times the interquartile range, where the quartiles were determined by applying the transformation given in Table S2 to the quartiles of the standard normally distributed variable *Z*_5_.

Finally, we generated the clustered binary outcome *Y* using the R-package ‘SimCorMultRes’ [23] assuming equal correlation between all pairs of outcomes within one cluster, corresponding to the assumption of an exchangeable working correlation structure. We required the outcome *Y* to satisfy *P*(*Y* = 1| *x*) = (1 + exp(*xβ*))^−1^, where *x* = (1, *x*_1_, …*x*_5_) is a realization of the covariates and *β* = (*β*_0_, *β*_1_, …*β*_5_) is the true vector of regression coefficients with *β*_1_, …*β*_5_ specified in Table S2 and *β*_0_ chosen such that the desired event rate was achieved. The package SimCorMultRes generates clustered categorical responses by sampling from a latent regression model with clustered continuous responses and then dichotomizing them by applying thresholds. In particular, the desired dependence structure is expressed in terms of the correlation matrix of the latent responses. We used correlation coefficients of 0.7 and 0.9 at the level of latent responses for the scenarios with moderate and high correlation, respectively. See Fig. S3 for the achieved correlation at the level of binary outcomes.

#### Methods

We analyzed each data set using generalized estimating equations (GEE, as implemented in the R package ‘mmmgee’), single-step augmented GEE (augGEE1), iterated augmented GEE (augGEE) and penalized GEE (pGEE), always applying an exchangeable working correlation structure. In addition, we considered single-step augmented GEE with independent working correlation structure (augGEE1, ind), which always gives finite coefficient estimates and served as a back-up method. To get a better understanding of the convergence behavior of augmented GEE, we also investigated single-step augmented GEE with the true coefficient estimates as starting values and single-step augmented GEE with the correlation parameter fixed at the true value with respect to convergence. For all estimators confidence intervals were calculated from the small-sample corrected [13] sandwich estimates of the variance-covariance matrix using the t-distribution. We used the R-package ‘detectseparation’ [24] to check for separation in the simulated data sets.

#### Estimands

The estimands in this study were the regression coefficients with special focus on the coefficient corresponding to our binary main variable of interest *X*_1_ with a true coefficient of 0.69, their standard errors and the predicted probabilities.

#### Performance measures

We assessed the methods’ rates of convergence by classifying a model fit as non-convergent if the fitting procedure declared non-convergence or resulted in an error, if the estimated correlation parameter was outside of (−1, 1) or if the absolute distance between one of the coefficient estimates ${\hat{\beta}}_j,j=1,\dots, p$ and its true value *β*_*j*_ was larger than ten times the square root of the *j*-th diagonal element of the sandwich variance estimate applied to the simulated data sets using the true coefficient estimates and the true working correlation matrix, averaged over all 1000 simulation runs. For the point estimate of *β*_1_ and predictions, we evaluated bias and root mean squared error (RMSE). The mean squared error of predictions was first calculated as ${\left({N}^{\ast}\right)}^{-1}\sum_{i=1}^{N^{\ast }}{\left({\hat{\mu}}_i-{\mu}_i\right)}^2$ for each data set, where ${\hat{\mu}}_i$ and *μ*_*i*_ are the estimated and true predicted probabilities for the *i*-th observation, respectively, and where *N*^∗^ is the number of observations, and then averaged over all generated data sets in a scenario. We report the root of this average as RMSE of predictions. For confidence intervals, we evaluated the coverage rates (nominal level 0.95) and power (probability to exclude 0). Whenever ordinary GEE, augmented GEE or penalized GEE did not converge for a data set we replaced the non-convergent fit by the results from single-step augmented GEE with independent working correlation structure for calculating the performance measures. We summarized the simulation results graphically using nested loop plots [25].

### Results

#### Convergence

**Fig. 2:**
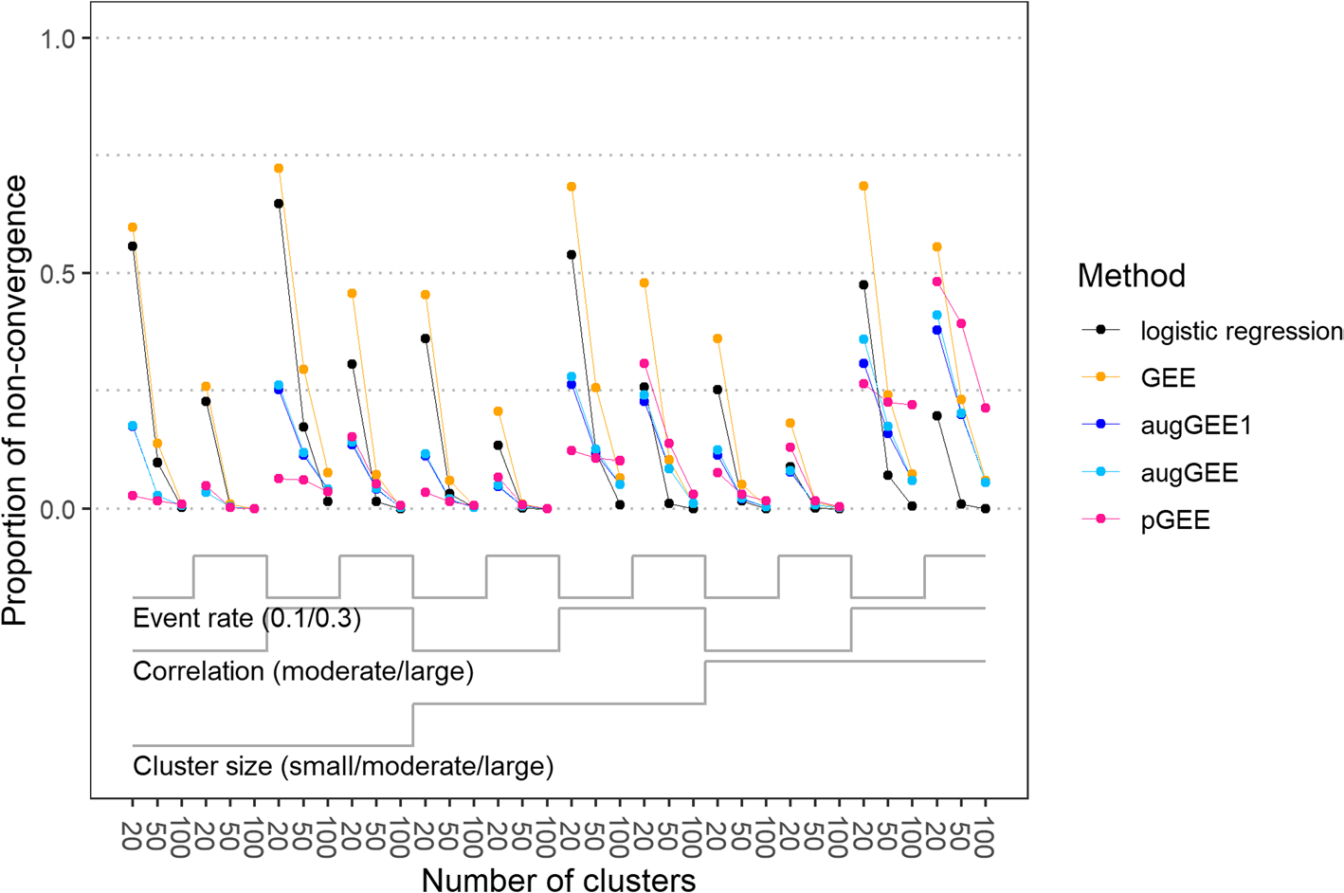
Nested loop plot for the proportion of non-convergence with logistic regression, generalized estimating equations (GEE), single-step augmented GEE (augGEE1), iterated augmented GEE (augGEE) and penalized GEE (pGEE) for the 36 scenarios. For scenarios with small, moderate or large cluster size, the numbers of observations per cluster were sampled from a truncated Poisson distribution with mean 5, 10 or 20, respectively. A moderate or large correlation refers to a correlation coefficient of 0.7 or 0.9 at the level of latent responses. ‘Event rate’ denotes the expected proportion of Y = 1 in a scenario

The largest proportion of separable data sets (0.65) was observed for the scenario with 20 clusters, small cluster size, highly correlated outcome and an event rate of 0.1, see Fig. 2. The proportion of data sets where GEE ran into convergence issues was always equal to or higher than the proportion of separable data sets. As expected, the single-step and the iterated augmented GEE approaches generally had fewer convergence issues than ordinary GEE. For instance, in the scenario with the highest number of separable data sets the proportion of non-convergence was only 0.25 and 0.26 for the single-step and the iterated augmented GEE, respectively, while it was 0.72 for GEE. Recall that single-step augmented GEE is performed by stopping the iterated augmented GEE algorithm after the first outer iteration and using the hat matrix $\overset{\sim }{H}$ for independent data instead of the one generalized to clustered data from the iterated augmented GEE algorithm. Thus, it is not surprising that the proportion of non-convergence for the single-step algorithm was never higher than for the iterated algorithm, but the differences were negligible. The proportion of non-convergence for penalized GEE was often even lower than the one for augmented GEE, especially in scenarios with 20 clusters and an event rate of 0.1, but was higher than the proportion of non-convergence for ordinary GEE in scenarios with 100 clusters, large cluster size and strong correlation.

Contrary to what was stated by Mondol and Rahman [4], penalized GEE as well as the augmented GEE approaches do not guarantee finite estimates of the regression coefficients in the presence of separation. Generally, a larger number of clusters, weaker correlation and a higher event rate were associated with a lower proportion of non-convergence.

Recall that we use the FL regression coefficients as starting values in the GEE fit performed in the single-step augmented GEE algorithm. This choice of starting values led to even better convergence behavior than using the true regression coefficients as starting values, which are not available in practice, see Fig. S2. The discrepancy between the proportions of convergence with the two choices of starting values underlines the importance of sensible starting values with GEE. Fixing the correlation parameter in single-step augmented GEE to the true value, another option not possible in practice, substantially improved the convergence behavior but still resulted in a proportion of non-convergence of 0.35 in the worst scenario, see Fig. S2.

#### Correlation

Figure S3 illustrates that the true correlation of the binary outcome did not only depend on the correlation of the latent continuous variables used by the R-package ‘SimCorMultRes’ [23] to generate the binary outcome but also on the event rate. The true correlation ranged from 0.39 for scenarios with moderate correlation of the latent variables and event rate of 0.1 to 0.67 for scenarios with high correlation of latent variables and event rate of 0.3. All methods tended to underestimate the true correlation but this effect was especially pronounced with ordinary GEE and penalized GEE when the data sets consisted of only 20 clusters and the event rate was small. Generally, the methods gave estimated correlation parameters closer to the true value if the number of clusters was larger.

#### Point estimates for regression coefficients

**Fig. 3:**
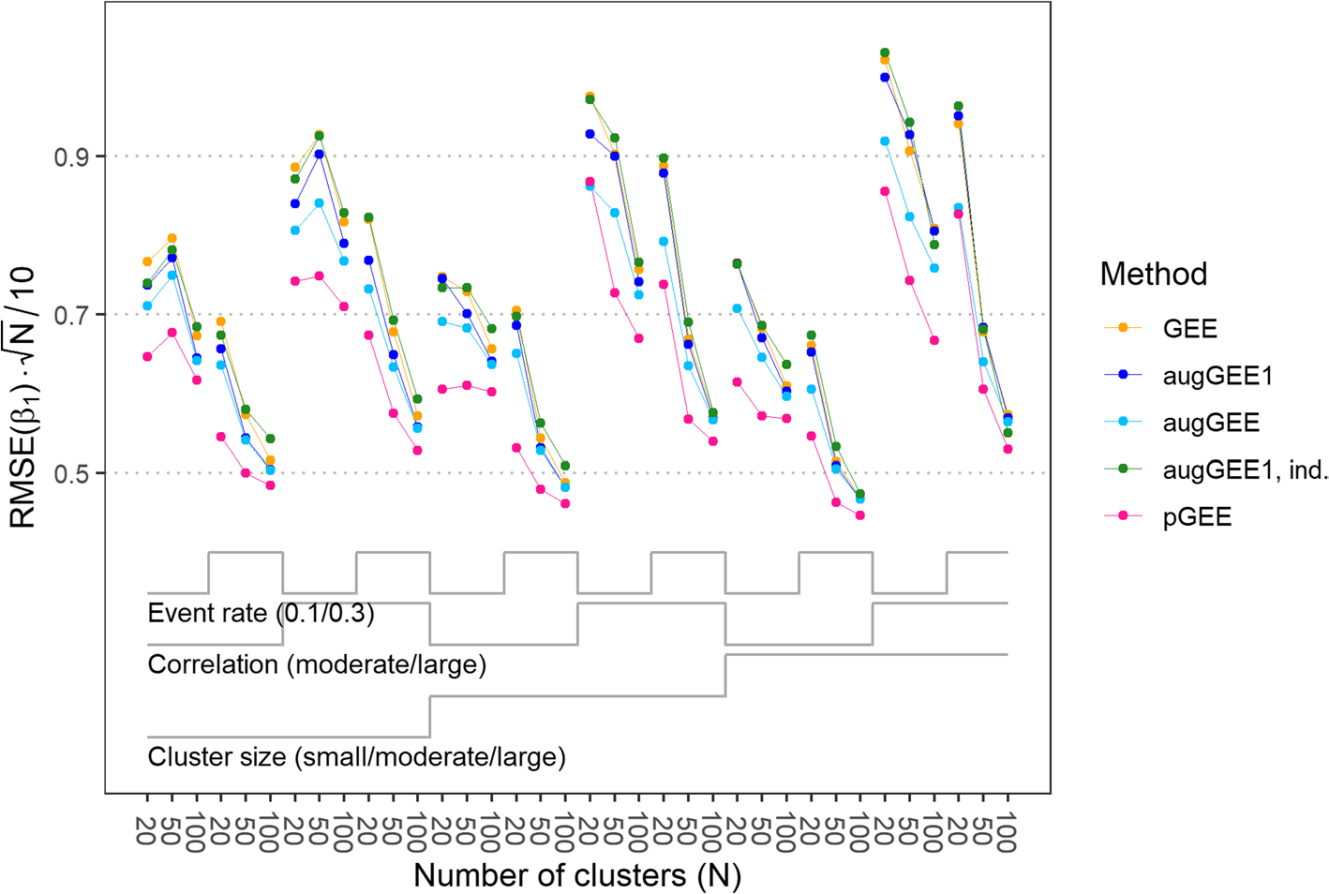
Root mean squared error (RMSE) of *β*_1_ multiplied by the square root of the number of clusters (*N*) divided by 10 with generalized estimating equations (GEE), single-step augmented GEE (augGEE1), iterated augmented GEE (augGEE), single-step augmented GEE with independent working correlation structure (augGEE1, ind) and penalized GEE (pGEE) for the 36 scenarios. Regression coefficient *β*_1_ corresponds to the binary main variable of interest with a true value of 0.69. In the calculation of the RMSE, non-convergent fits by ordinary GEE, augmented GEE or penalized GEE were replaced by the results from single-step augmented GEE with independent working correlation structure. For scenarios with small, moderate or large cluster size, the numbers of observations per cluster were sampled from a truncated Poisson distribution with mean 5, 10 or 20, respectively. A moderate or large correlation refers to a correlation coefficient of 0.7 or 0.9 at the level of latent responses. ‘Event rate’ denotes the expected proportion of Y = 1 in a scenario

Single-step and iterated augmented GEE almost always gave estimates for the five regression coefficients of lower RMSE than ordinary GEE but the differences were small, cf. Figure 3 for the results for *β*_1_ and Fig. S4 for the results for the other coefficients. Penalized GEE was the method which most often resulted in the smallest RMSE. As expected, the RMSE of the regression coefficients was smaller for scenarios with larger number of clusters and higher event rate. We could not identify a systematically superior behavior of any estimator in regard to the bias of regression coefficients, see Fig. S5 for the results for *β*_1_. Generally, the bias was small compared to the RMSE.

#### Confidence intervals for regression coefficients

**Fig. 4:**
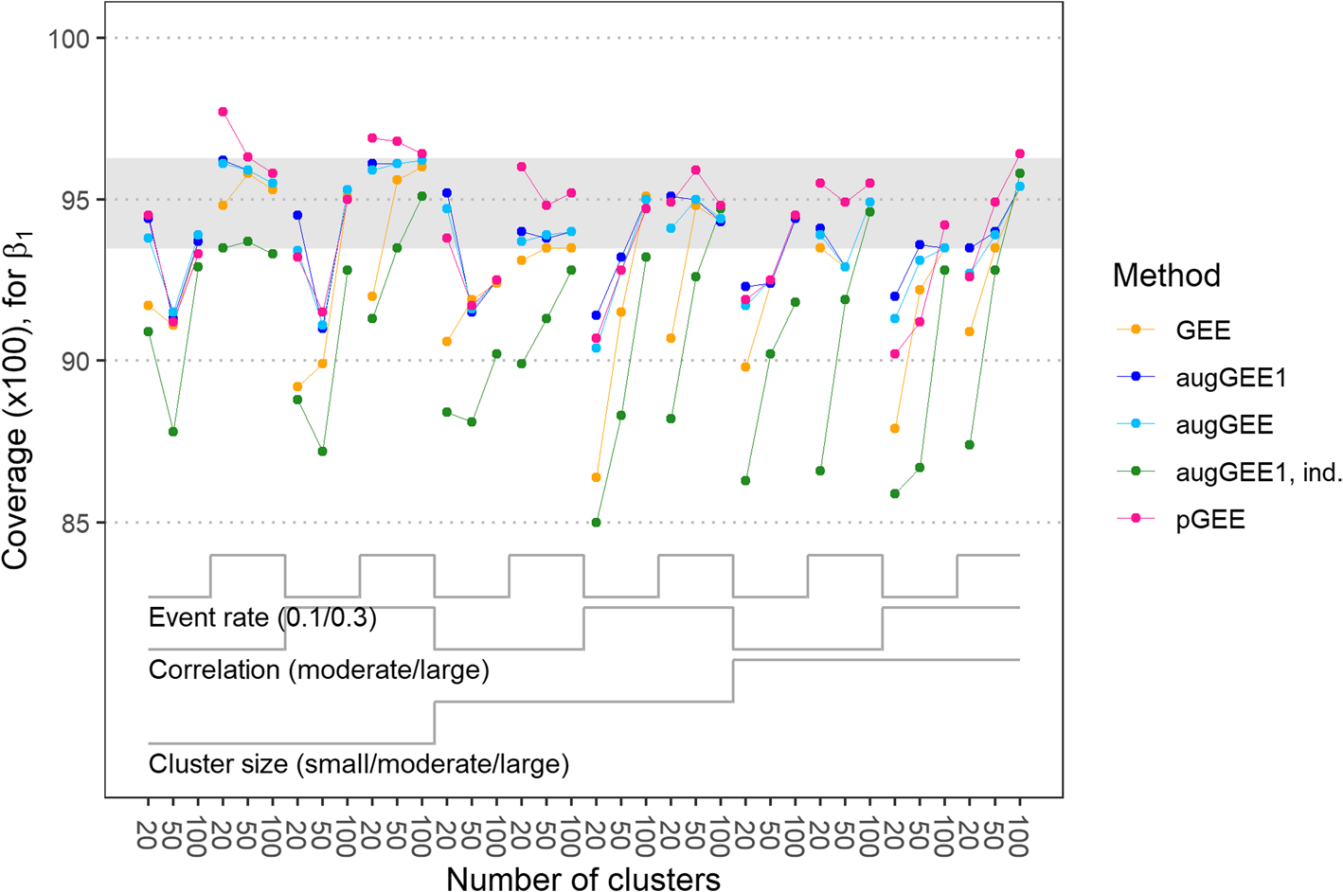
Coverage of 95% confidence intervals for *β*_1_ with generalized estimating equations (GEE), single-step augmented GEE (augGEE1), iterated augmented GEE (augGEE), single-step augmented GEE with independent working correlation structure (augGEE1, ind) and penalized GEE (pGEE) for the 36 scenarios. The coverage was calculated as the relative frequency of data sets where the confidence intervals included the true regression coefficient *β*_1_ = 0.69. In the calculation of the coverage, non-convergent fits by ordinary GEE, augmented GEE or penalized GEE were replaced by the results from single-step augmented GEE with independent working correlation structure. The grey band (0.935 to 0.963) represents the Monte Carlo error (95 % confidence interval) at an observed probability of 0.95 with 1000 repetitions. For scenarios with small, moderate or large cluster size, the numbers of observations per cluster were sampled from a truncated Poisson distribution with mean 5, 10 or 20, respectively. A moderate or large correlation refers to a correlation coefficient of 0.7 or 0.9 at the level of latent responses. ‘Event rate’ denotes the expected proportion of Y = 1 in a scenario

Applying the small-sample correction to the sandwich-covariance estimates as proposed by Morel et al. [13] substantially improved the performance of all considered estimators in terms of coverage of the confidence intervals. However, the 95 % confidence intervals still were often too anti-conservative for our binary main variable of interest *X*_1_, especially for the single-step augmented GEE with independent working correlation structure, see Fig. 4 for the overall coverage and Fig. S6 for the left- and right-tailed coverage. For the other four covariates the results were similar, see Fig. S7. For the sake of completeness Fig. S8 shows the power of the 95 % confidence intervals but this measure is difficult to interpret when actual coverage probabilities were lower than the nominal level.

#### Predictions

**Fig. 5:**
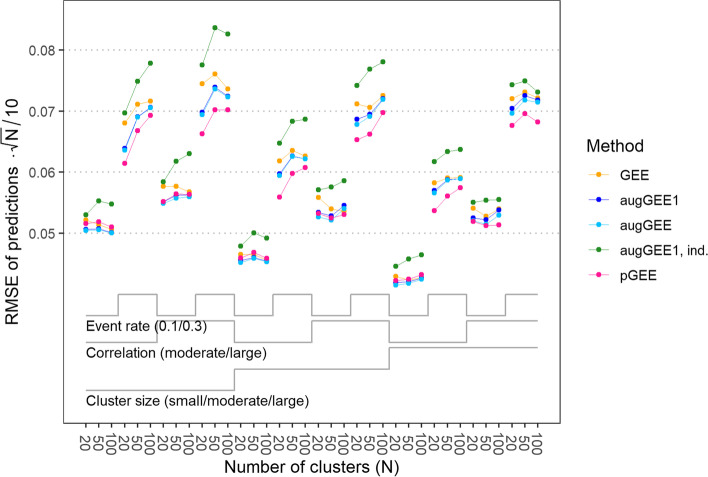
Root mean squared error (RMSE) of predictions multiplied by the square root of the number of clusters (*N*) divided by 10 with generalized estimating equations (GEE), single-step augmented GEE (augGEE1), iterated augmented GEE (augGEE), single-step augmented GEE with independent working correlation structure (augGEE1, ind) and penalized GEE (pGEE) for the 36 scenarios. In the calculation of the RMSE, non-convergent fits by ordinary GEE, augmented GEE or penalized GEE were replaced by the results from single-step augmented GEE with independent working correlation structure. For scenarios with small, moderate or large cluster size, the numbers of observations per cluster was sampled from a truncated Poisson distribution with mean 5, 10 or 20, respectively. A moderate or large correlation refers to a correlation coefficient of 0.7 or 0.9 at the level of latent responses. ‘Event rate’ denotes the expected proportion of Y = 1 in a scenario

Similarly as for the regression coefficients, the augmented GEE approaches and penalized GEE performed better than ordinary GEE with regard to the RMSE of predictions in 35 and 30, respectively, out of the 36 simulation scenarios, see Fig. 5. Ignoring the correlation of the binary outcome by assuming an independent working correlation structure with single-step augmented GEE resulted in predicted probabilities of larger RMSE than with any other method for all scenarios.

Logistic regression with independent data gives predicted probabilities that sum up to the number of events [7]. This property does not transfer to the situation of clustered data: in our simulations, ordinary GEE tended to overestimate event rates for scenarios with an event rate of 0.1 and lower number of clusters, see Fig. S9. This tendency for overestimation was even more severe for penalized GEE, single-step and iterated augmented GEE. One might suspect that the overestimation of predicted probabilities by ordinary GEE observed in Fig. S9 could be an artefact caused by our strategy of replacing non-convergent fits by results from single-step augmented GEE with independent working correlation structure for the calculation of the RMSE of prediction. However, calculating the RMSE only from the convergent fits gave very similar results.

## Discussion

In this paper we have investigated methods that transfer FL to GEE. In addition to the evaluation of penalized GEE, a recently published procedure, we proposed to transfer FL to GEE by exploiting the equivalence of FL to maximum likelihood estimation with an augmented data set. Under independence, penalized GEE as well as single-step and iterated augmented GEE give the same coefficient estimates as FL. Moreover, they substantially improve convergence compared to ordinary GEE according to our simulations, while showing a solid performance in terms of accuracy of coefficient estimates and predictions. As there was little difference in the performance between the two augmented GEE approaches, for practical purposes we recommend the simpler one, the single-step augmented GEE. It can be easily implemented in any statistical software where implementations of FL and weighted ordinary GEE are available, see Box S1 for a worked example using R code. Moreover, the idea of augmented GEE could be easily transferred to other settings, for instance to clustered count data analyzed by marginal Poisson regression models. While in our simulations the performance of penalized GEE often was slightly superior to the performance of the augmented GEE approaches, a major drawback is the burden of implementation. Penalized GEE transfers FL to GEE by treating the GEE as score equations and requires fundamental modifications of the GEE fitting algorithm. Thus, data analysts who are interested in applying penalized GEE but are not willing to spend a considerable amount of time and effort in coding are reliant on available software implementations, which up to now are scarce. To the best of our knowledge there is only one implementation of penalized GEE, which is based on R and is available on GitHub. We provide a modified version of this implementation (https://github.com/heogden/geefirthr) which fixes some minor issues such as the handling of clusters consisting of only one observation. In summary, for fitting marginal logistic regression models on sparse data we recommend to use penalized GEE if one has access to a suitable software implementation and single-step augmented GEE otherwise.

The idea to augment the data by pseudo data with balanced outcome in order to improve convergence of GEE was already touched upon by Woods and van de Ven [26] in an engineering context. They proposed to give equal weight to the pseudo observations, while with our approach the weights of the pseudo observations are set proportional to the diagonal elements of the generalized hat matrix. In the approach by Woods and van de Ven there is no canonical choice of the number of added pseudo observations and they called for future research to tackle this issue before the method can be applied in practice.

An early attempt to improve the convergence behavior of GEE is the ‘one-step GEE algorithm’ proposed by Lipsitz et al. [27], which is obtained by performing only a single step of the ordinary GEE algorithm using the regression coefficients from logistic regression as starting values. One shortcoming of this one-step estimator is that it only exists if logistic regression gives finite coefficient estimates, i.e. if the data are non-separable, but this could be easily overcome by using regression coefficients from FL as starting values. However, while the idea of the one-step estimator sounds convincingly simple, in practice users face the problem that the various available implementations of GEE (e.g. geeglm in the R package ‘geepack’, gee in the package ‘gee’) give different solutions when being stopped after one iteration. Admittedly, the authors specified the one-step GEE algorithm in detail but left it to the users either to start coding themselves or to check which, if any, of the existing GEE implementations result in the required algorithm when being stopped after one iteration.

While non-convergence in logistic regression is widely understood and can be appealingly characterized in terms of simple properties of the data set using the concept of separation, the issue is more complicated with GEE. For instance, to the best of our knowledge, it has not even been proven yet that GEE do not give finite coefficient estimates for separable data sets as suggested by simulations. With GEE, the non-convergence is not always related to coefficient estimates diverging to infinity during model fitting but can also be related to a correlation structure approaching singularity [27]. It is unclear whether a simple characterization of the data sets where GEE do not converge can be given, paralleling the concept of separation for logistic regression. Interestingly, we observed that omitting observations might cause GEE to converge even when the estimator does not converge on the full data set, a phenomenon which cannot occur for logistic regression.

FL can be modified to give average predicted probabilities equal to the observed event rate by using the data augmentation representation and adding a binary covariate that is equal to 0 for the original observations and equal to 1 for the pseudo observations [7]. This modification, called FL with added covariate (FLAC) by Puhr et al., could be straightforwardly applied to the two augmented GEE approaches. However, we have not pursued this approach further as some preliminary simulations indicated that the small reduction in the bias of predicted probabilities might come at the cost of a slightly increased RMSE of coefficient estimates and more non-convergence issues.

In this paper we focused on the example of clustered data where an exchangeable working correlation structure is the most plausible one, but all the considered methods can handle any correlation structure. However, future research is needed to investigate the performance of the augmented GEE approaches with correlation structures other than exchangeable. In general, all three extensions of FL to GEE will have to prove themselves in applications and comprehensive simulation studies, especially as they lack strict theoretical foundation.

## Supplementary Information

**Additional file 1.**

## Data Availability

Our modified version of the R package ‘geefirthr’, which can also deal with clusters containing one observation and with the scale parameter set to 1, can be found at https://github.com/heogden/geefirthr. R code for the single-step and iterated augmented GEE can be found at https://github.com/AngelikaGeroldinger/augGEE. The data of the implant dentistry study can be found in Joshi et al. [27]

## Abbreviations

augGEE: Iterated augmented generalized estimating equations
augGEE1: Single-step augmented generalized estimating eqs.
FL: Firth’s logistic regression
GEE: Generalized estimating equations
pGEE: Penalized generalized estimating equations
RMSE: Root mean squared error
