# Supplementary material for: An investigation of penalization and data augmentation to improve convergence of generalized estimating equations for clustered binary outcomes
Source: BMC Med Res Methodol. 2022 Jun 9;22:168. doi: 10.1186/s12874-022-01641-6 (PMC9178839; doi:10.1186/s12874-022-01641-6)

An investigation of penalization and data augmentation to improve convergence of generalized estimating equations for clustered binary outcomes

Angelika Geroldinger, Rok Blagus, Helen Ogden and Georg Heinze

Supplementary File

Box S1. Example R code for performing single-step augmented GEE with small-sample corrected standard errors based on the sandwich estimator applied to the augmented data, relating the binary outcome $Y.bin$ with the two explanatory variables $x1$ and $x2$ from the data set datasim, which comes with the package ‘mmmgee’.

library(Matrix)

library(logistf)
library(mmmgee)

data(datasim)

# fit a FL model to get the hat matrices for the weights of the augmented data set;
fit_FL <- logistf(Y.bin ~ x1 + x2, data=datasim, firth=TRUE, pl=FALSE)

# create the augmented data set;
data_aug <- data.frame(rbind(datasim, datasim, datasim))
# reverse the outcome in the third copy of original data;
data_aug$Y.bin <- c(datasim$Y.bin, datasim$Y.bin, 1- datasim$Y.bin)
# create the weights;
data_aug$weights <- c(rep(1, nrow(datasim)), 0.5*fit_FL$hat.diag, 0.5*fit_FL$hat.diag)
# create the cluster id for the augmented data set;
data_aug$id <- c(datasim$id, datasim$id + max(datasim$id) , datasim$id + 2*max(datasim$id))

# GEE on the augmented data;
fit <- geem2(formula=Y.bin ~ x1 + x2, family = binomial("logit"), id = id, data = data_aug, weights=weights, scale.fix=TRUE, corstr="exchangeable", useP=FALSE)

# small-sample corrected variance matrix according to Morel, 2003;
nstar <- nrow(fit$X)/3 # number of observations in the original data set;
Y <- fit$y[1:nstar] # original outcome;
X <- fit$X[1:nstar,] # original design matrix;
k <- ncol(X) # number of parameters including intercept;
id <- fit$sandw.args$id[1:nstar] # original cluster id variable;
n <- length(unique(id[1:nstar])) # number of clusters in the original data set;
R.alpha.inv <- fit$sandw.args$R.alpha.inv[1:nstar, 1:nstar] # correlation structure for the original data;
mu <- (1+exp(-fit$sandw.args$eta[1:nstar]))^-1 # predicted probabilities in the original data;
w <- as.vector(mu*(1-mu))
hess <- Matrix::crossprod(diag(sqrt(w)) %*% X, R.alpha.inv %*% diag(sqrt(w)) %*% X) # Hessian matrix for the original data;
hessinv <- solve(as.matrix(hess))
d <- sapply(unique(id), function(x) t(Y[id==x]- mu[id==x]) %*% diag(1/sqrt(w[id==x])) %*% R.alpha.inv[id==x,id==x, drop=FALSE] %*% diag(sqrt(w[id==x])) %*% X[id==x,, drop=FALSE])
d_bar <- Reduce("+", d)/n
I1 <- sapply(d, function(x) t(x-d_bar) %*% (x-d_bar))
I1 <- Reduce('+', I1)
mult <- ((nstar-1)/(nstar-k)) * (n/(n-1))
phi <- max(1, sum(diag(mult * as.matrix(hessinv %*% I1))) /k)
delta <- min(0.5, k/(n-k))
sw <- I1 %*% hessinv
sw <- hessinv %*% sw # sandwich variance matrix;
swcor <- as.matrix( mult* sw + delta * phi * hessinv) # small-sample corrected sandwich variance matrix;

summary(fit)

## Estimate Model SE Robust SE Wald p
## (Intercept) 0.018841 0.2630 0.2619 0.07193 0.9427
## x1 0.002195 0.3078 0.2793 0.00786 0.9937
## x2 -0.360315 0.2616 0.2522 -1.42850 0.1531
##
## Estimated Correlation Parameter: 0.4582
## Correlation Structure: exchangeable
## Fixed Scale Parameter: 1
##
## Number of GEE iterations: 4
## Number of Clusters: 120 Maximum Cluster Size: 4
## Number of observations with nonzero weight: 390

# small-sample corrected standard errors:
sqrt(diag(swcor))

## (Intercept) x1 x2
## 0.2839421 0.3069806 0.2756751

Figure S1. Implant dentistry study: stacked bar plot of number of implants with and without haematological complications per patient. The data set includes 533 implantations in edentulous jaws, performed in 134 patients.


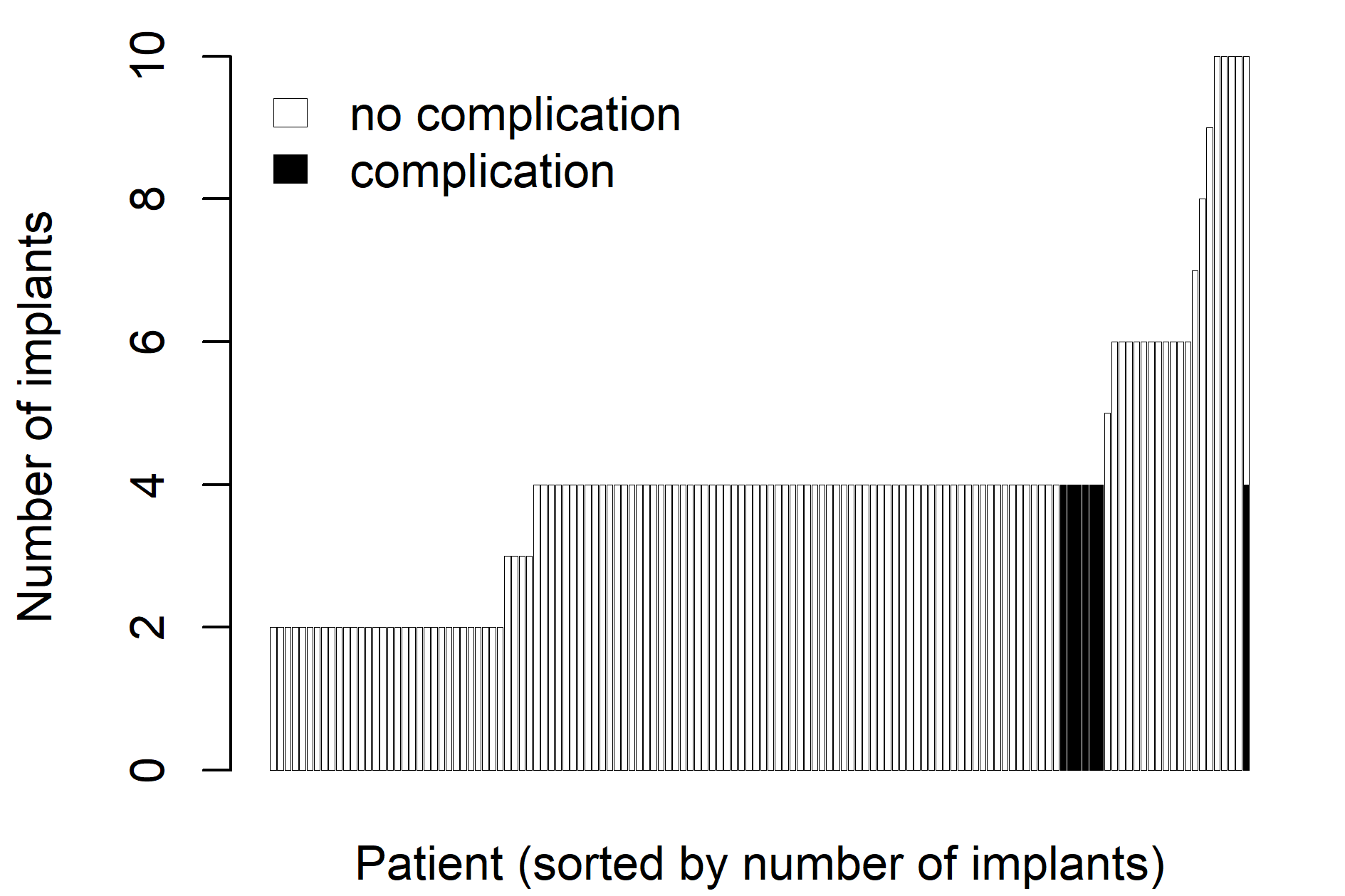


Table S1. Implant dentistry study: regression coefficients (95% confidence intervals) for the association of haematological complications with timing of implant placement (immediate/early/later), diabetes mellitus (yes/no), antiresorptive therapy (yes/no) and age (in decades) estimated with multivariable GEE (geeglm function in the R package ‘geepack’), single-step augmented generalized estimating equations (augGEE1), iterated augmented GEE (augGEE) and penalized GEE (pGEE). 95% confidence intervals were calculated from the small sample corrected sandwich variance-covariance matrix for augGEE1, augGEE and pGEE and from the usual sandwich variance-covariance matrix for geeglm.

|  | Timing (immediate vs late) | Timing  (early vs late) | Diabetes M. | Antiresorptive therapy | Age  (decades) |
| --- | --- | --- | --- | --- | --- |
| geeglm | 0.51  (-0.77, 1.79) | -0.03  (-0.32, 0.26) | 1.73  (0.1, 3.35) | -39.03  (-40.3, -37.76) | 0.04  (-0.57, 0.64) |
| augGEE1 | 0.71  (-0.43, 1.86) | 0.04  (-0.24, 0.32) | 1.68  (0.15, 3.21) | -0.12  (-1.46, 1.22) | 0.02  (-0.5, 0.54) |
| augGEE | 0.64  (-0.49, 1.78) | 0.06  (-0.22, 0.34) | 1.66  (0.14, 3.18) | -0.42  (-1.81, 0.96) | 0.02  (-0.51, 0.56) |
| pGEE | 0.67  (-0.49, 1.82) | 0.02  (-0.3, 0.34) | 1.69  (0.23, 3.16) | 0.01  (-1.32, 1.34) | 0.03  (-0.5, 0.55) |

Table S2. Simulation study: information on the generation of the covariates $X_{1},\ldots X_{5}$, including pairwise correlations between the standard normally distributed underlying variables $Z_{1},\ldots Z_{5}$, the transformations defining $X_{k}$, the measurement scale, the expected value and the true coefficients for $X_{k}$. $I$ denotes the indicator function.

|  | Pairwise correlation with | | | | | Constant within cluster | Transformation defining $X_{k}$ | Scale of $X_{k}$ | $E(X_{k})$ | $\beta_{k}$ |
| --- | --- | --- | --- | --- | --- | --- | --- | --- | --- | --- |
|  | $Z_{1}$ | $Z_{2}$ | $Z_{3}$ | $Z_{4}$ | $Z_{5}$ |  |  |  |  |  |
| $Z_{1}$ | $1$ | $0.5$ | $0.5$ |  |  | yes | $X_{1}=I(Z_{1}<-1)$ | binary | $0.16$ | $0.69$ |
| $Z_{2}$ | $0.5$ | $1$ |  | $0.5$ |  | yes | $X_{2}=I(Z_{2}<-1)$ | binary | 0.16 | $0.69$ |
| $Z_{3}$ | $0.5$ |  | $1$ |  | $-0.3$ | no | $X_{3}=I(Z_{3}<0)$ | binary | 0.5 | $0.69$ |
| $Z_{4}$ |  | $0.5$ |  | $1$ | $0.5$ | no | $X_{4}=I\left( Z_{4}\geq0.5 \right)+I(Z_{4}\geq1.5)$ | ordinal | 0.38 | $0.35$ |
| $Z_{5}$ |  |  | $-0.3$ | $0.5$ | $1$ | no | $X_{5}=10 Z_{5}+55$ | metric | 55 | $-0.035$ |

Figure S2. Simulation study: nested loop plot for the proportion of non-convergence with logistic regression, single-step augmented GEE (augGEE1), single-step augmented GEE with the true coefficients as starting values (augGEE1 – true coef. as start) and single-step augmented GEE with the correlation parameter fixed at the true value (augGEE1 – fixed corr.) for the 36 scenarios. For scenarios with small, moderate or large cluster size, the numbers of observations per cluster were sampled from a truncated Poisson distribution with mean 5, 10 or 20, respectively. A moderate or large correlation refers to a correlation coefficient of 0.7 or 0.9 at the level of latent responses. 'Event rate' denotes the expected proportion of Y=1 in a scenario.


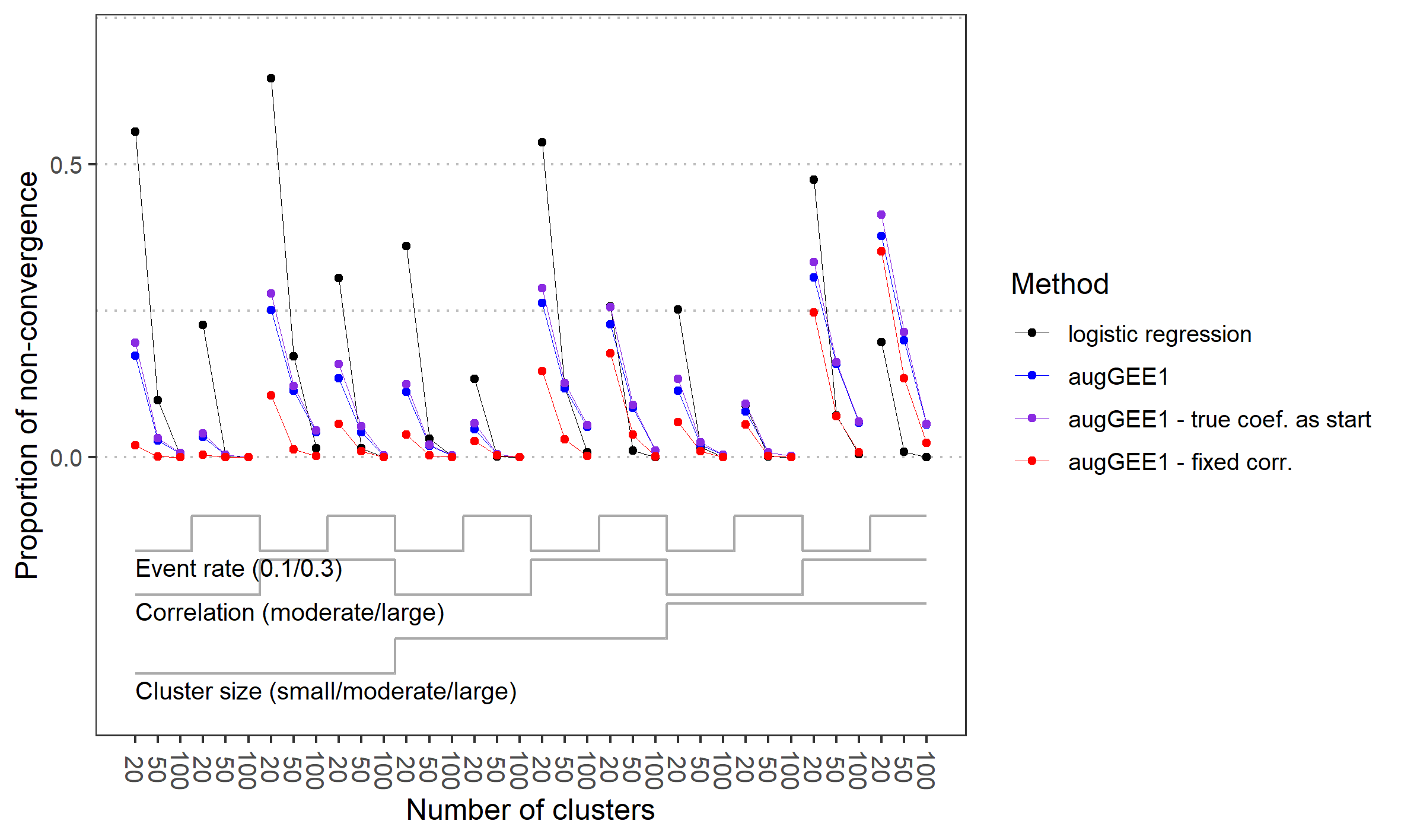


Figure S3. Simulation study: mean within-cluster correlation of the binary outcome for the $36$ scenarios, estimated with generalized estimating equations (GEE), single-step augmented GEE (augGEE1), iterated augmented GEE (augGEE) and penalized GEE (pGEE). The ‘true correlation’ for a certain event rate, correlation (of the underlying latent variables) and cluster size was approximated by generating one large data set with 100,000 clusters and applying ordinary GEE. In calculating the mean correlation for a certain estimator we restricted to the data sets where the estimator converged. For scenarios with small, moderate or large cluster size, the numbers of observations per cluster were sampled from a truncated Poisson distribution with mean 5, 10 or 20, respectively. A moderate or large correlation refers to a correlation coefficient of 0.7 or 0.9 at the level of latent responses. 'Event rate' denotes the expected proportion of Y=1 in a scenario.


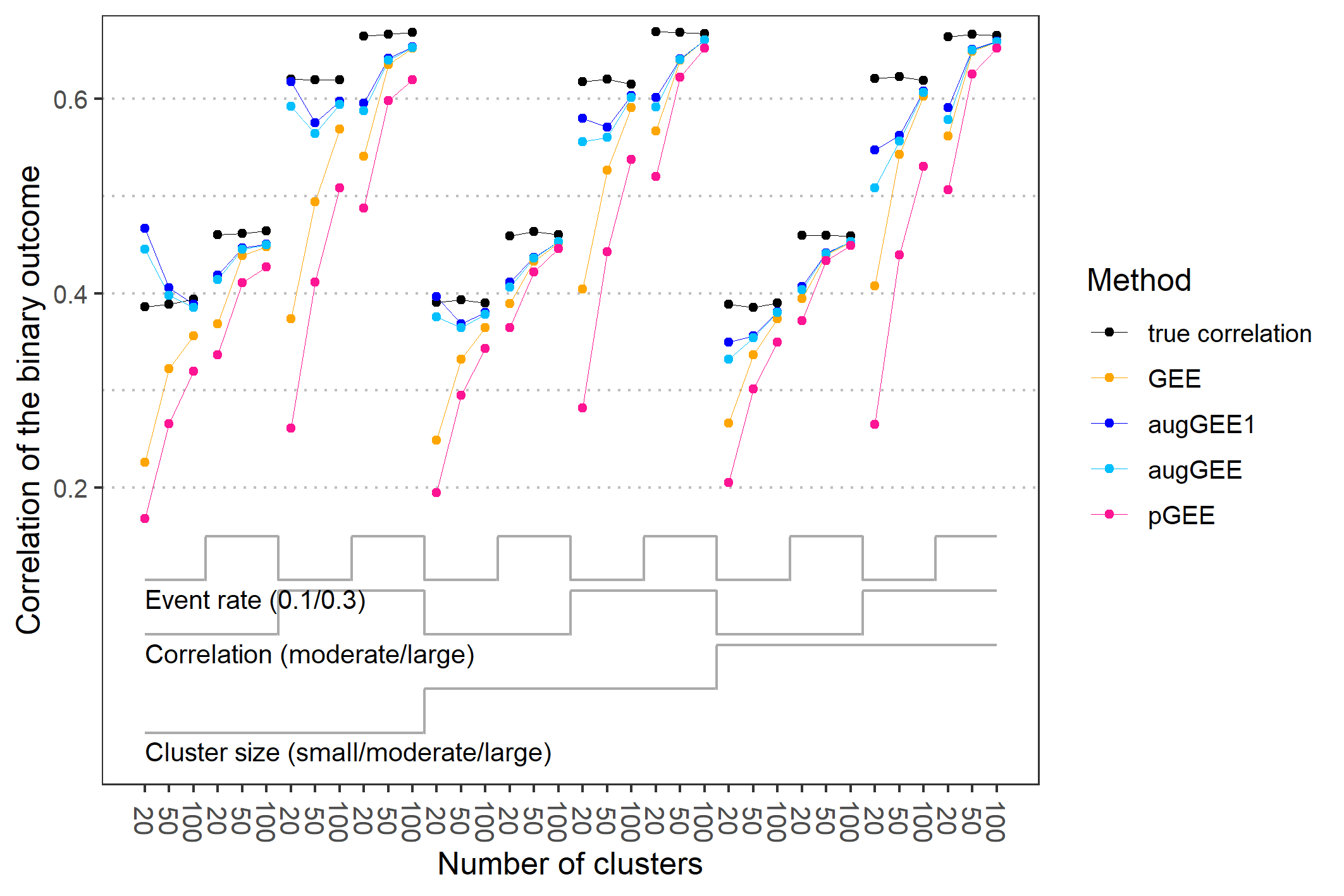


Figure S4. Root mean squared error (RMSE) of $\beta_{2},\beta_{3}, \beta_{4}$ and $\beta_{5}$ multiplied by the square root of the number of clusters ($N$) divided by $10$ with generalized estimating equations (GEE), single-step augmented GEE (augGEE1), iterated augmented GEE (augGEE), single-step augmented GEE with independent working correlation structure (augGEE1, ind) and penalized GEE (pGEE) for the $36$scenarios. In the calculation of the RMSE, non-convergent fits by ordinary GEE, augmented GEE or penalized GEE were replaced by the results from single-step augmented GEE with independent working correlation structure. For scenarios with small, moderate or large cluster size, the numbers of observations per cluster were sampled from a truncated Poisson distribution with mean $5, 10$ or $20$, respectively. A moderate or large correlation refers to a correlation coefficient of $0.7$ or $0.9$ at the level of latent responses. 'Event rate' denotes the expected proportion of Y=1 in a scenario.


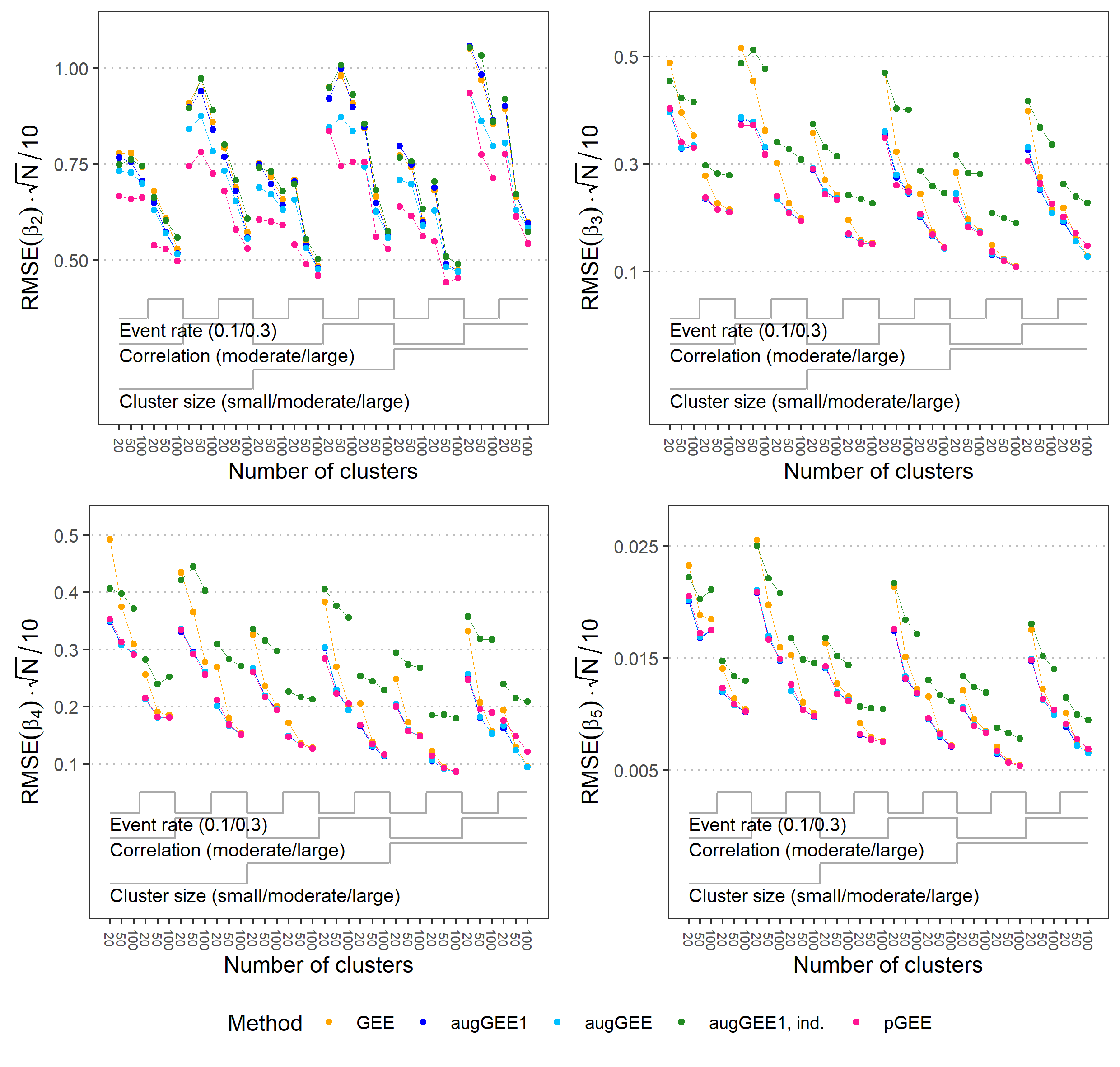


Figure S5. Simulation study: bias with generalized estimating equations (GEE), single-step augmented GEE (augGEE1), iterated augmented GEE (augGEE), single-step augmented GEE with independent working correlation structure (augGEE1, ind) and penalized GEE (pGEE) for the $36$ scenarios. In the calculation of the bias, non-convergent fits by ordinary GEE, augmented GEE or penalized GEE were replaced by the results from single-step augmented GEE with independent working correlation structure. For scenarios with small, moderate or large cluster size, the numbers of observations per cluster were sampled from a truncated Poisson distribution with mean $5, 10$ or $20$, respectively. A moderate or large correlation refers to a correlation coefficient of $0.7$ or $0.9$ at the level of latent responses. 'Event rate' denotes the expected proportion of Y=1 in a scenario.


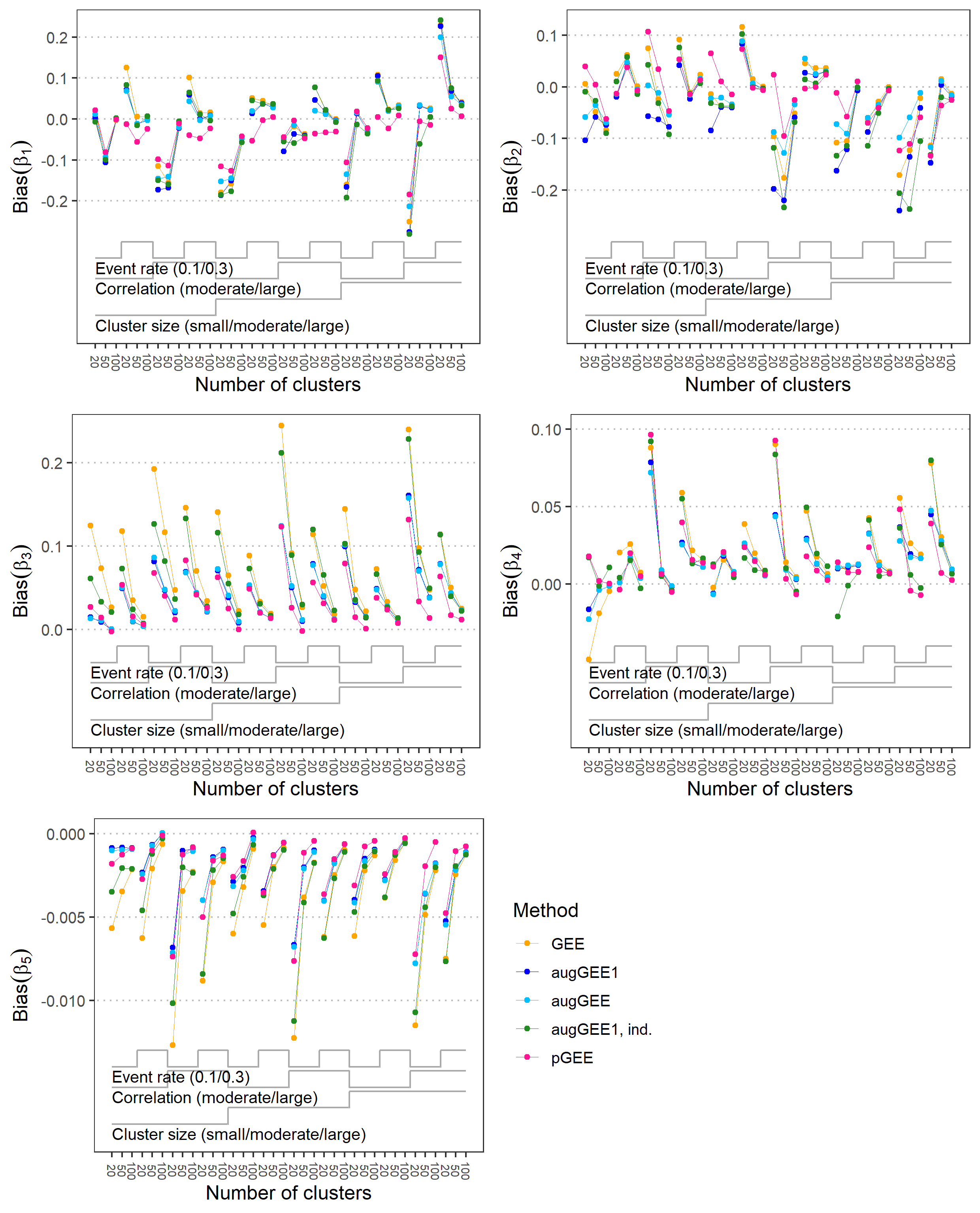


Figure S6. Simulation study: left- and right-tailed coverage of the $95$% confidence intervals for $\beta_{1}$ with generalized estimating equations (GEE), single-step augmented GEE (augGEE1), iterated augmented GEE (augGEE), single-step augmented GEE with independent working correlation structure (augGEE1, ind) and penalized GEE (pGEE) for the $36$ scenarios. The coverage was calculated as the proportion of data sets where the confidence intervals included the true regression coefficient $\beta_{1}=0.69$. In the calculation of the coverage, non-convergent fits by ordinary GEE, augmented GEE or penalized GEE were replaced by the results from single-step augmented GEE with independent working correlation structure. The grey band ($0.963$ to $0.984$) represents the Monte Carlo error ($95$% confidence interval) at an observed probability of $0.95$ with $1000$ repetitions. For scenarios with small, moderate or large cluster size, the numbers of observations per cluster were sampled from a truncated Poisson distribution with mean $5, 10$ or $20$, respectively. A moderate or large correlation refers to a correlation coefficient of $0.7$ or $0.9$ at the level of latent responses. 'Event rate' denotes the expected proportion of Y=1 in a scenario.
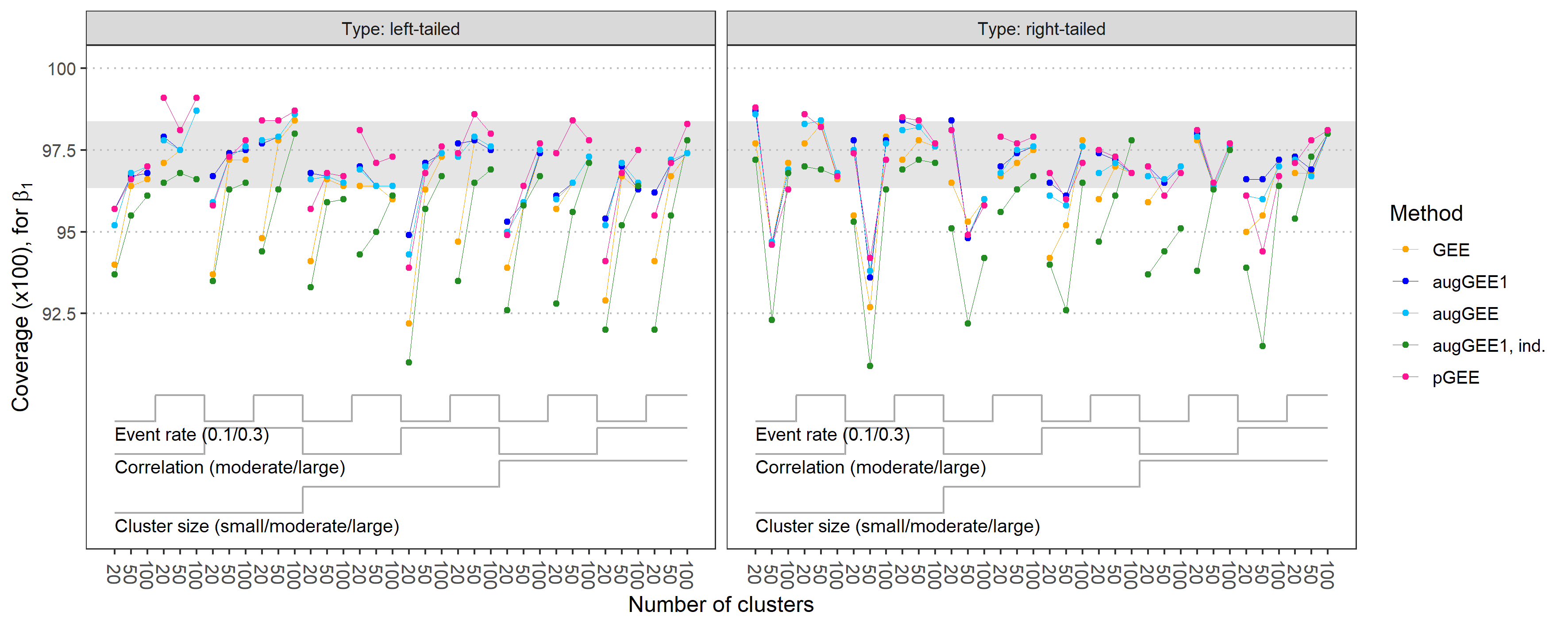


Figure S7. Simulation study: coverage of the $95$% confidence intervals for the intercept and the five explanatory variables with generalized estimating equations (GEE), single-step augmented GEE (augGEE1), iterated augmented GEE (augGEE), single-step augmented GEE with independent working correlation structure (augGEE1, ind) and penalized GEE (pGEE) for the $36$ scenarios. The coverage was calculated as the proportion of data sets where the confidence intervals included the true regression coefficient. In the calculation of the coverage, non-convergent fits by ordinary GEE, augmented GEE or penalized GEE were replaced by the results from single-step augmented GEE with independent working correlation structure. The grey band ($0.935$ to $0.963$) represents the Monte Carlo error ($95$% confidence interval) at an observed probability of $0.95$ with $1000$ repetitions. For scenarios with small, moderate or large cluster size, the numbers of observations per cluster were sampled from a truncated Poisson distribution with mean $5, 10$ or $20$, respectively. A moderate or large correlation refers to a correlation coefficient of $0.7$ or $0.9$ at the level of latent responses. 'Event rate' denotes the expected proportion of Y=1 in a scenario.
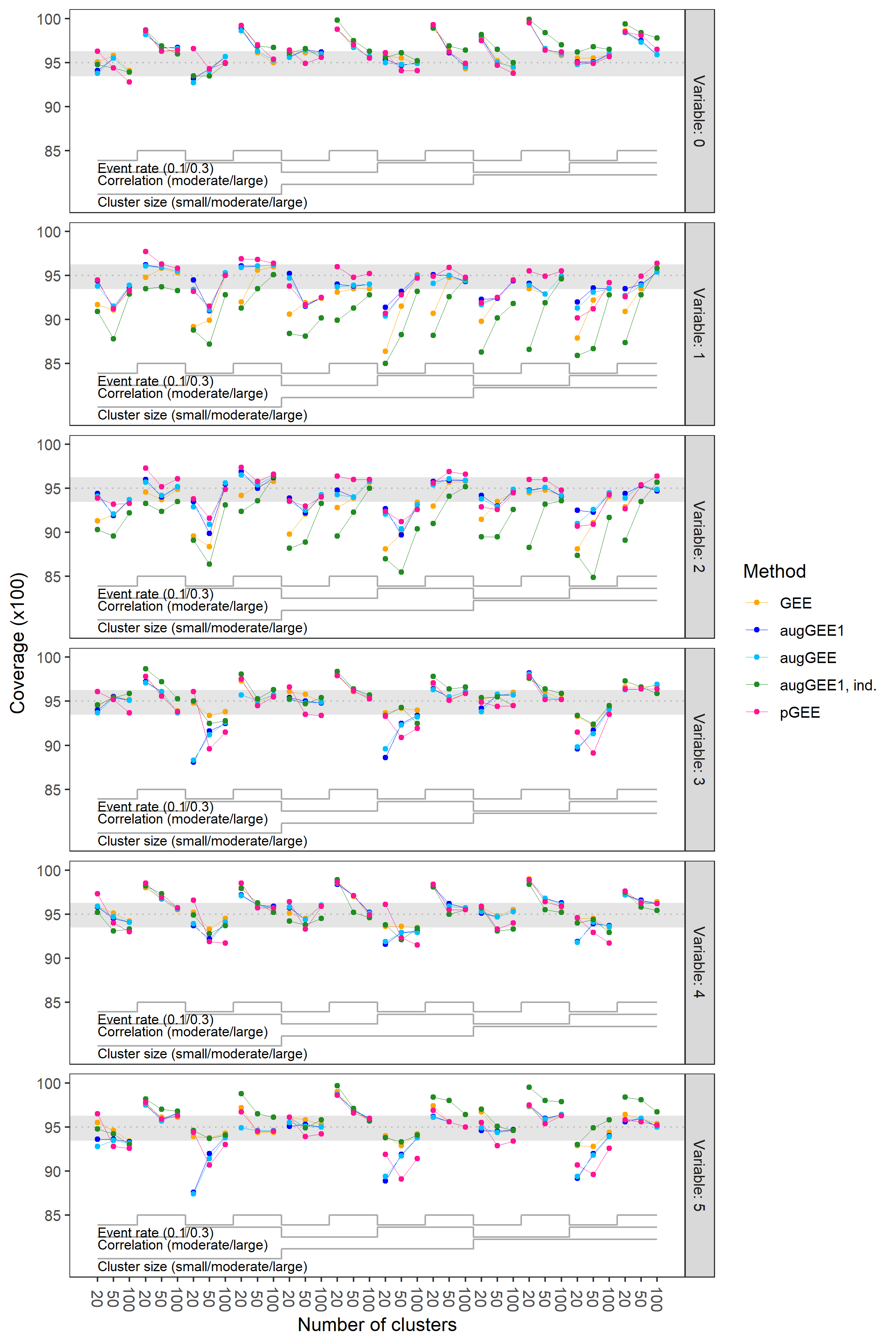


Figure S8. Simulation study: power of the $95$% confidence intervals for $\beta_{1}$ with generalized estimating equations (GEE), single-step augmented GEE (augGEE1), iterated augmented GEE (augGEE), single-step augmented GEE with independent working correlation structure (augGEE1, ind) and penalized GEE (pGEE) for the $36$ scenarios. The power was calculated as the proportion of data sets where the confidence intervals excluded $0$. In the calculation of the power, non-convergent fits by ordinary GEE, augmented GEE or penalized GEE were replaced by the results from single-step augmented GEE with independent working correlation structure. For scenarios with small, moderate or large cluster size, the numbers of observations per cluster were sampled from a truncated Poisson distribution with mean $5, 10$ or$20$, respectively. A moderate or large correlation refers to a correlation coefficient of $0.7$ or $0.9$ at the level of latent responses. 'Event rate' denotes the expected proportion of Y=1 in a scenario.


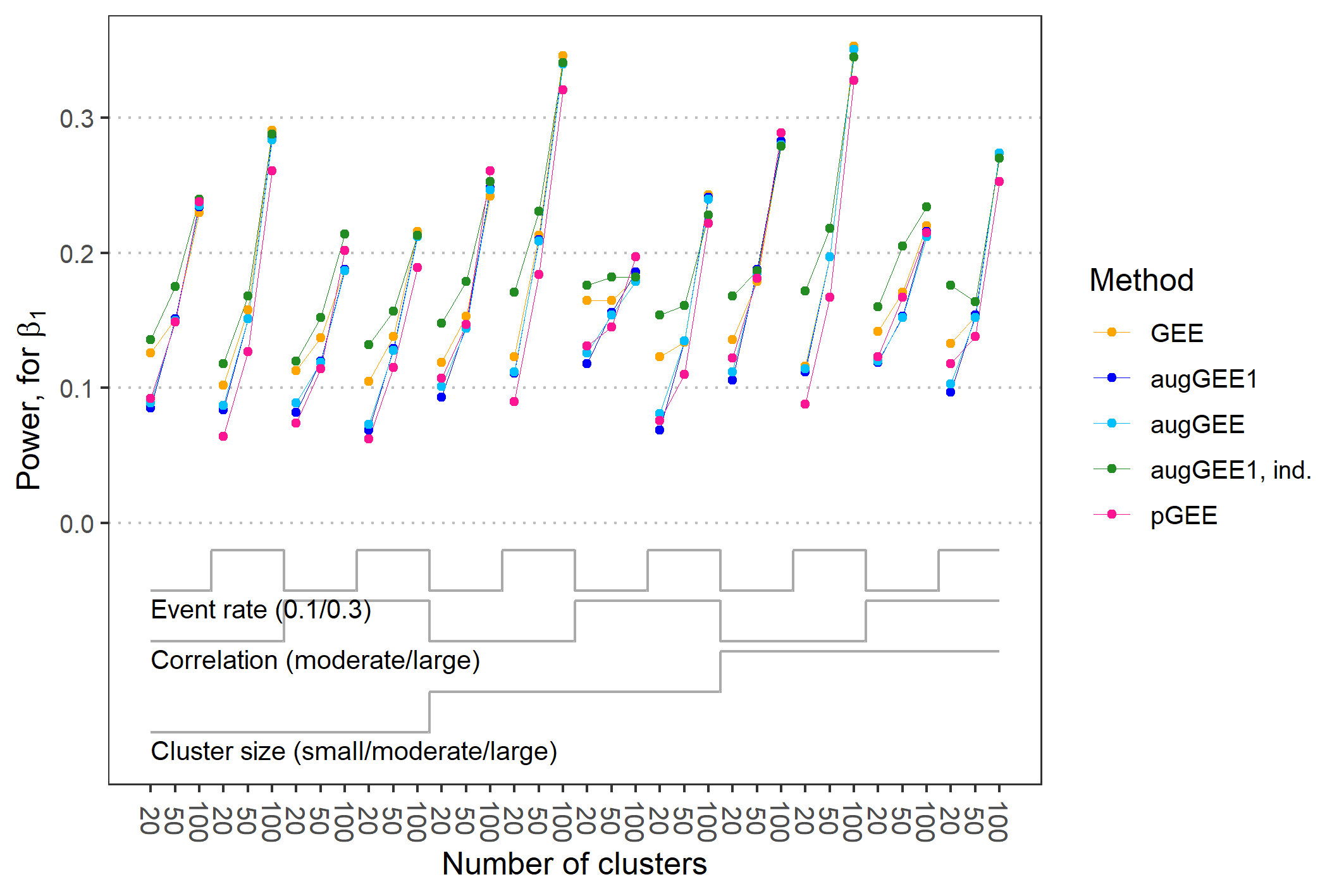


Figure S9. Simulation study: bias of predictions with generalized estimating equations (GEE), single-step augmented GEE (augGEE1), iterated augmented GEE (augGEE), single-step augmented GEE with independent working correlation structure (augGEE1, ind) and penalized GEE (pGEE) for the $36$ scenarios. In the calculation of the bias, non-convergent fits by ordinary GEE, augmented GEE or penalized GEE were replaced by the results from single-step augmented GEE with independent working correlation structure. For scenarios with small, moderate or large cluster size, the numbers of observations per cluster were sampled from a truncated Poisson distribution with mean $5$, $10$ or $20$, respectively. A moderate or large correlation refers to a correlation coefficient of $0.7$ or $0.9$ at the level of latent responses. 'Event rate' denotes the expected proportion of Y=1 in a scenario.


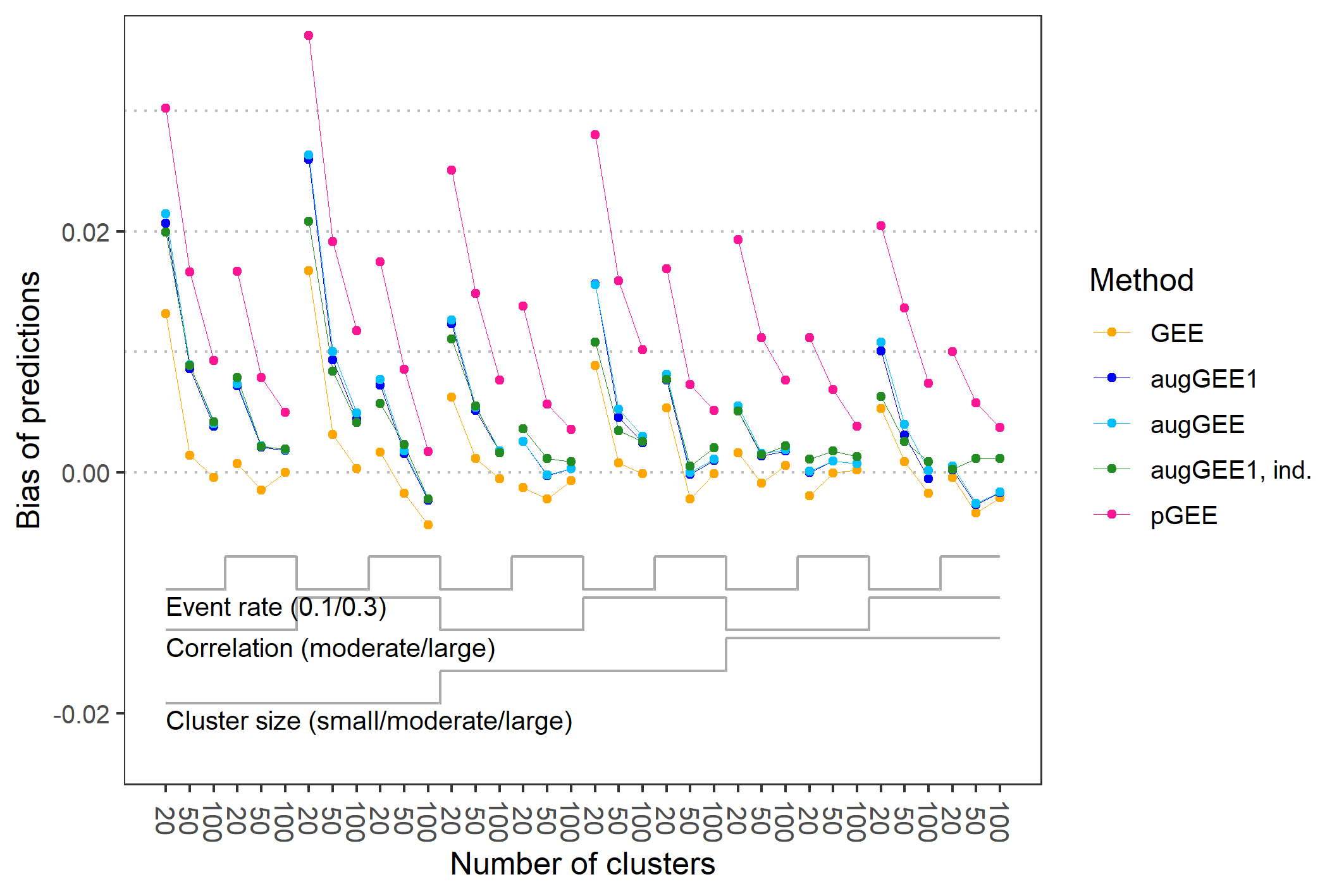

Supplement: Supplementary file 1 — Additional file 1. [file 12874_2022_1641_MOESM1_ESM.docx]
